# Myeloid DRP1 deficiency limits revascularization in ischemic muscles via inflammatory macrophage polarization and metabolic reprogramming

**DOI:** 10.1172/jci.insight.177334

**Published:** 2025-01-09

**Authors:** Shikha Yadav, Vijay C. Ganta, Sudhahar Varadarajan, Vy Ong, Yang Shi, Archita Das, Dipankar Ash, Sheela Nagarkoti, Malgorzata McMenamin, Stephanie Kelley, Tohru Fukai, Masuko Ushio-Fukai

**Affiliations:** 1 Vascular Biology Center,; 2 Department of Medicine (Cardiology), and; 3 Department of Pharmacology and Toxicology, Medical College of Georgia at Augusta University, Augusta, Georgia, USA.; 4 Charlie Norwood Veterans Affairs Medical Center, Augusta, Georgia, USA.; 5 Biostatistics and Bioinformatics Core, Karmanos Cancer Institute, Department of Oncology, Wayne State University School of Medicine, Detroit, Michigan, USA.

**Keywords:** Angiogenesis, Inflammation, Macrophages

## Abstract

Macrophages play a crucial role in promoting perfusion recovery and revascularization after ischemia through antiinflammatory polarization, a process essential for the treatment of peripheral artery disease (PAD). Mitochondrial dynamics, particularly regulated by the fission protein DRP1, are closely linked to macrophage metabolism and inflammation. However, the role of DRP1 in reparative neovascularization remains unexplored. Here, we show that DRP1 expression was increased in F4/80^+^ macrophages within ischemic muscle on day 3 after hind limb ischemia (HLI), an animal model of PAD. Mice lacking *Drp1* in myeloid cells exhibited impaired limb perfusion recovery, angiogenesis, and muscle regeneration after HLI. These effects were associated with increased proinflammatory M1-like macrophages, p-NF-κB, and TNF-α, and reduced antiinflammatory M2-like macrophages and p-AMPK in ischemic muscle of myeloid *Drp1*^–/–^ mice. In vitro, *Drp1*-deficient macrophages under hypoxia serum starvation (HSS), an in vitro PAD model, demonstrated enhanced glycolysis via reducing p-AMPK as well as mitochondrial dysfunction, and excessive mitochondrial ROS production, resulting in increased proinflammatory M1-gene and reduced antiinflammatory M2-gene expression. Conditioned media from HSS-treated *Drp1*^–/–^ macrophages exhibited increased proinflammatory cytokine secretion, leading to suppressed angiogenesis in endothelial cells. Thus, macrophage DRP1 deficiency under ischemia drives proinflammatory metabolic reprogramming and macrophage polarization, limiting revascularization in experimental PAD.

## Abstract 2

Drp1 deficiency in macrophages under ischemia drives pro-inflammatory macrophage polarization and metabolic reprogramming via suppressing p-AMPK and inducing excess mitochondrial ROS, which limits reparative neovascularization.

## Graphical abstract

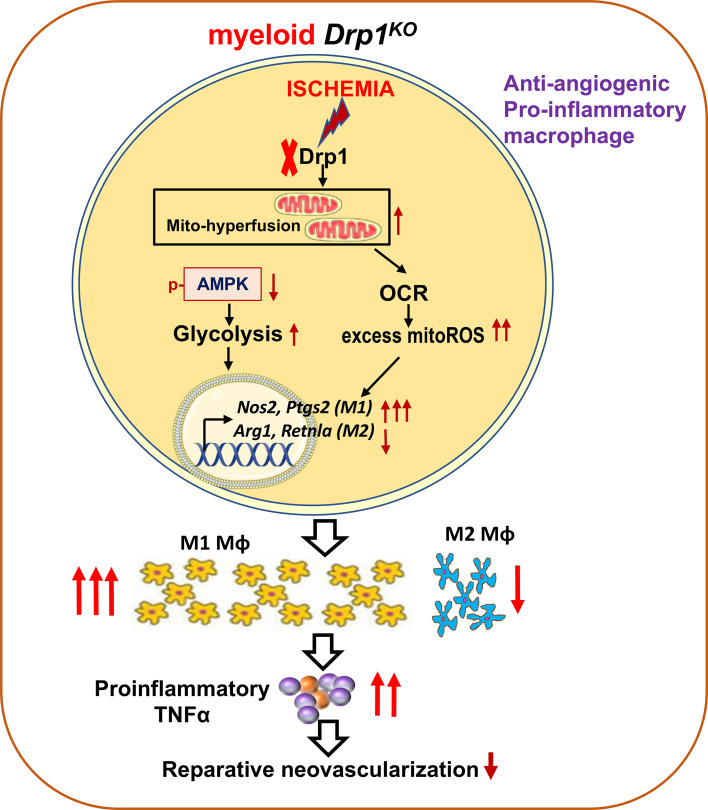

## Introduction

Peripheral artery disease (PAD) poses a substantial burden of morbidity and mortality due to tissue damage resulting from acute and chronic occlusive ischemia (1, 2). Angiogenesis is the formation of new blood vessels from pre-existing ones, which requires endothelial cells (ECs) to proliferate, migrate, and differentiate to form new vascular structures and is indispensable for restoring limb perfusion of PAD (3). Therapeutic angiogenesis has faced multiple disappointments, with clinical trials failing to demonstrate a difference in primary endpoint effects (4). Macrophages play an important role in restoring perfusion recovery and revascularization following ischemia via antiinflammatory polarization and producing angiogenic factors, which is required for treatment of PAD (5–9). In the early phases of the response to ischemic injury, a hypoxic and proinflammatory environment attracts proinflammatory M1-type macrophages characterized by enhanced aerobic glycolytic metabolism. Subsequently, a metabolic transition occurs, shifting macrophage phenotypes toward antiinflammatory M2-type macrophages with increased oxidative phosphorylation in mitochondria, thus promoting reparative angiogenesis and neovascularization (10–13). Macrophage phenotype transition is a complex process influenced by tissue-specific and stimulus-specific differentiation, activation, and maturation programs (14). Defects in normal macrophage metabolism and polarization can lead to unresolved tissue injury, and exacerbation of tissue damage and impaired healing processes (10, 11). The critical process of macrophage metabolic reprogramming for tissue repair is in part dependent on mitochondrial metabolism and dynamics (15); however, the underlying mechanisms in the context of ischemia-induced neovascularization are poorly understood.

Mitochondria are highly dynamic organelles that orchestrate metabolic and immune adaptations in response to ischemia. Prolonged ischemia can compromise cellular bioenergetics due to mitochondrial dysfunction (16). Mitochondrial dynamics, continuously adapting to extracellular stimuli, are tightly regulated by mitochondrial fission proteins, including dynamin-related GTPase DRP1 and mitochondrial fission factor 1 (MFF1), and mitochondrial fusion proteins, including mitofusins (MFN1and MFN2) and ortic atrophy1 (OPA1). Recent reports suggest the role of mitochondrial dynamics in regulating mitochondrial reactive oxygen species (mitoROS) levels, calcium homeostasis, and oxidative phosphorylation (17). Altered mitochondrial dynamics in macrophages are a universal response to a variety of inflammatory stimuli. In a context-dependent fashion, DRP1 plays a role in regulating pro- or antiinflammatory macrophage phenotypes (18–22). It has been shown that the inhibition of DRP1 using Mdivi-1 or *Drp1* siRNA transfection leads to a reduction in glycolysis, ROS production, and NF-κB–dependent proinflammatory M1 macrophage polarization when stimulated with lipopolysaccharide (LPS) (20). By contrast, DRP1 deficiency exacerbates LPS-induced systemic inflammation and liver injury in an in vivo murine model (19). However, the role of macrophage DRP1 in reparative neovascularization during ischemia in an experimental PAD has not been reported.

In the present study using myeloid-specific *Drp1*-knockout (*M*ɸ*Drp1*^KO^) mice with hindlimb ischemia (HLI), an animal model of PAD, we provide evidence that *M*ɸ*Drp1*^KO^ mice exhibit impaired blood flow recovery, neovascularization, and muscle regeneration in response to HLI. This was associated with increased proinflammatory M1-like macrophage polarization, p-NF-κB, and TNF-α, and decreased antiinflammatory M2-like macrophage polarization and phosphorylation of AMP-activated protein kinase (p-AMPK) in ischemic muscle. In vitro studies using bone marrow–derived macrophages (BMDMs) from wild-type (WT) and *M*ɸ*Drp1*^KO^ mice exposed to hypoxia serum starvation (HSS) showed that *Drp1*^KO^ BMDMs under HSS had enhanced glycolysis via reducing p-AMPK. These cells also exhibited a decreased mitochondrial oxygen consumption rate (OCR) and excessive mitoROS production, which in turn increased M1-genes and decreased M2-genes, and impaired angiogenic responses in cultured ECs. Our study revealed macrophage DRP1 as a positive regulator and therapeutic target for promoting reparative neovascularization by enhancing antiinflammatory macrophage polarization and metabolic reprogramming under ischemia.

## Results

### Myeloid DRP1 deficiency impairs reparative neovascularization via inhibiting angiogenesis and arteriogenesis in response to HLI.

**Figure 1:**
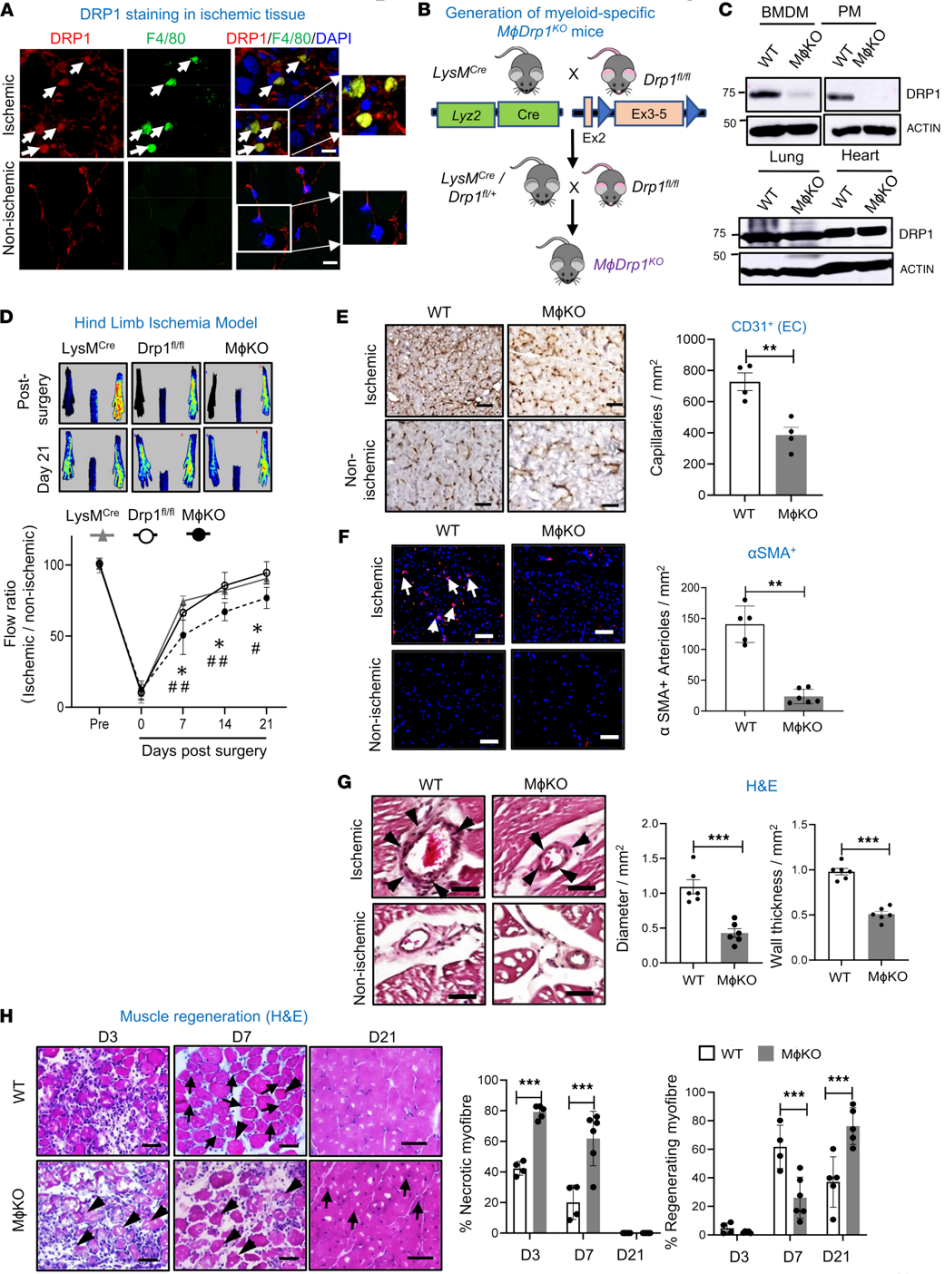
Myeloid *Drp1*^KO^ mice exhibited impaired reparative neovascularization via reducing angiogenesis and arteriogenesis in response to ischemia. (**A**) Immunofluorescence analysis for DRP1 (red) or F4/80^+^ macrophage (green) expression and their colocalization in non-ischemic and ischemic gastrocnemius (GC) muscles on day 3 after HLI. Scale bars: 10 μm. Inset shows region of interest at the same magnification. (**B**) Schematic representation of breeding strategy for generating myeloid-specific *Drp1^–/–^* (*M*ɸ*Drp1*^KO^) mice by crossing *LysM-*Cre mice with *Drp1^fl/fl^* mice. (**C**) Immunoblotting (IB) for DRP1 protein expression in bone marrow-derived macrophages (BMDMs), peritoneal macrophages (PMs), lungs, and hearts isolated from *Drp1^fl/fl^* (WT) and *M*ɸ*Drp1*^KO^ (MɸKO) mice. (**D**) Upper panels show representative laser Doppler images of legs on day 0 and day 21. Lower panels show the blood flow recovery after HLI as determined by the ratio of foot perfusion between ischemic (left) and non-ischemic (right) legs in *Drp1^fl/fl^*, *LysM-*Cre, and MɸKO mice (*n* = 8–12 mice per group, 2-way ANOVA followed by Tukey’s multiple-comparison test). *P < 0.05 for MɸDKO vs. *Drp1^fl/fl^* (WT); ^#^*P* < 0.05, ^##^*P* < 0.01 for MɸDKO vs. *LysM*-Cre^+/–^ *Drp1^+/+^* (WT). (**E**) Immunohistochemical analysis for CD31 staining (capillary density), *n* = 4 mice per group, unpaired, 2-tailed Student’s *t* test. (**F**) Immunofluorescence analysis of αSMA^+^ staining (arterioles) in ischemic and non-ischemic GC muscles in WT and MɸKO mice on day 21 after HLI. Scale bars: 20 μm. *n* = 5–6 mice per group, unpaired, 2-tailed Student’s *t* test. The right panel shows quantification. (**G**) H&E staining of ischemic and non-ischemic adductor (AD) muscles in WT and MɸKO mice on day 7 after HLI. Arrowheads show collateral arteries. Scale bars: 50 μm. The right panels show quantification of diameter and wall thickness of collateral arteries (unpaired, 2-tailed Student’s *t* test). (**H**) H&E staining of ischemic and non-ischemic GC muscles in WT and MɸKO mice on day 3 (D3), D7, and D21 after HLI. Scale bars: 20 μm. The right panels show quantification of percentage of necrotic and regenerating myofiber in these muscles (2-way ANOVA followed by Bonferroni’s multiple-comparison test). Data are mean ± SEM. *n* = 4–6. *P < 0.05; **P < 0.01; ***P < 0.001.

To assess the role of DRP1 in reparative neovascularization, we used the mouse HLI model that induces ischemia by femoral artery ligation and excision (23). Immunofluorescence (IF) analysis revealed that DRP1 was highly expressed in F4/80^+^ macrophages in ischemic muscles versus non-ischemic muscles on day 3 after HLI (Figure 1A). To determine the role of macrophage DRP1 in post-ischemic neovascularization, we generated *M*ɸ*Drp1*^KO^ mice by crossing homozygous *Drp1^fl/fl^* mice with mice expressing Cre recombinase under control of the lysozyme M promoter (*LysM*-Cre^+/–^) (Figure 1B). Selective deletion of DRP1 in macrophages was confirmed by protein analysis in BMDMs and peritoneal macrophages, but not in the lungs or hearts isolated from *M*ɸ*Drp1*^KO^ and WT mice (Figure 1C). Using laser speckle contrast perfusion imaging, we found no difference in blood flow recovery following HLI between *LysM*-Cre^+/–^ *Drp1^+/+^* WT and *LysM*-Cre^–/–^ *Drp1^fl/fl^* WT mice. In contrast, *M*ɸ*Drp1*^KO^ mice exhibited a reduction in blood flow recovery by day 21 after HLI compared with both WT groups (Figure 1D). Immunohistochemical analysis revealed that *M*ɸ*Drp1*^KO^ mice had fewer CD31-positive capillary-like ECs (Figure 1E) and α-smooth muscle actin–positive (αSMA-positive) arterioles (Figure 1F) in the ischemic gastrocnemius (GC) muscles compared with WT mice. However, there were no differences in CD31-positive ECs or αSMA-positive arterioles between the *LysM*-Cre^+/–^ *Drp1^+/+^* and *LysM*-Cre^–/–^ *Drp1^fl/fl^* WT groups (Supplemental Figure 1, A and B; supplemental material available online with this article; https://doi.org/10.1172/jci.insight.177334DS1). H&E staining showed that the increase in collateral lumen and wall area in semimembranous muscle induced by HLI was reduced in *M*ɸ*Drp1*^KO^ mice (Figure 1G). This was associated with increased necrotic myofibers and delayed muscle regeneration in ischemic muscles of *M*ɸ*Drp1*^KO^ mice versus WT mice (Figure 1H). Thus, macrophage DRP1 is required for restoring limb perfusion and promoting neovascularization by enhancing angiogenesis, arteriogenesis, tissue repair, and protection against necrosis in ischemic muscles following HLI.

### Myeloid DRP1 deficiency increases proinflammatory M1-like macrophages and decreases antiinflammatory M2-like macrophages without affecting the recruitment of Ly6C^hi^ monocytes in ischemic muscles.

**Figure 2:**
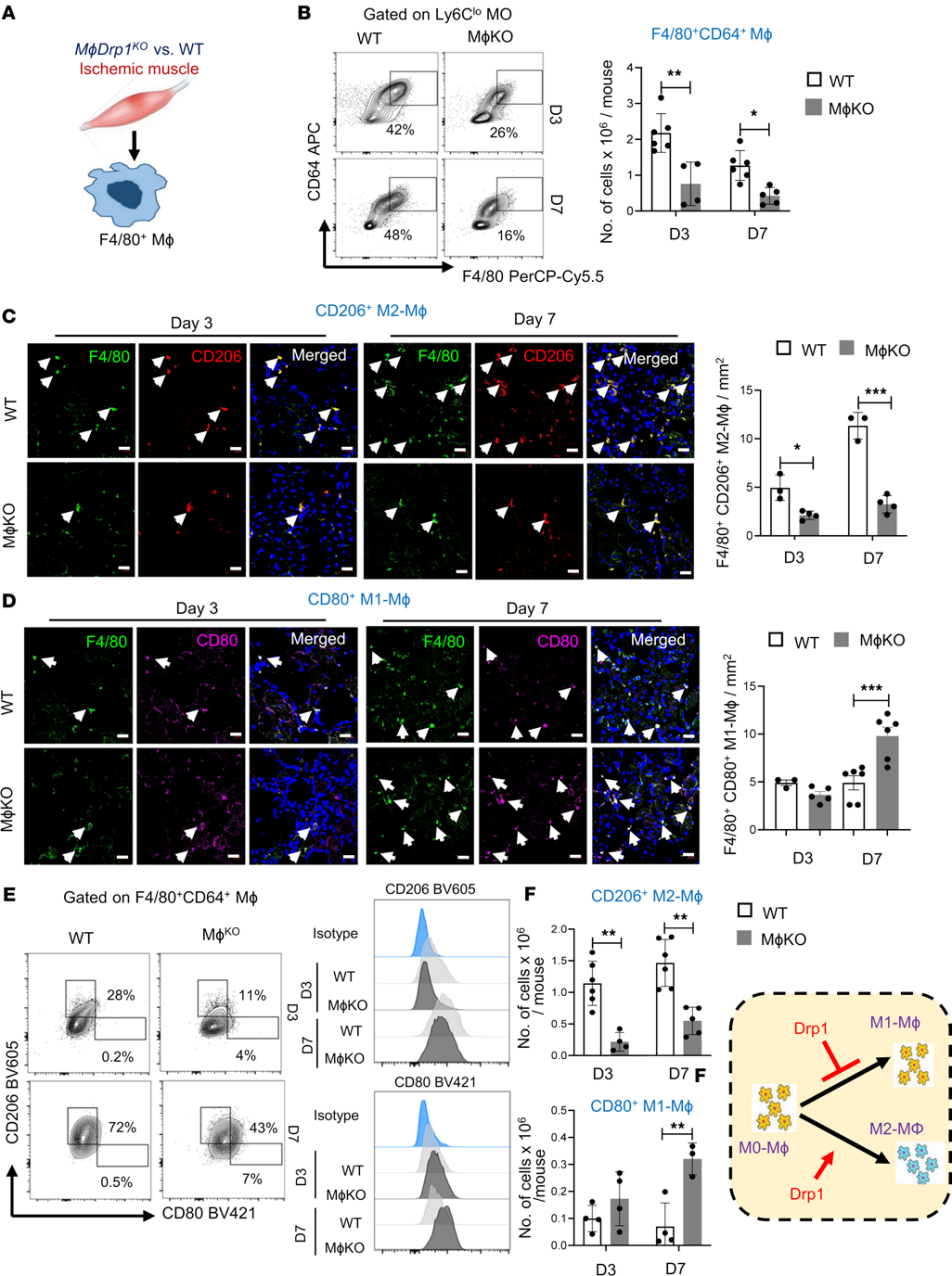
Myeloid *Drp1*^KO^ mice exhibited increase in proinflammatory M1-like macrophages and decrease in antiinflammatory M2-like macrophages in ischemic muscles. (**A**) Scheme showing FACS-based F4/80^+^ macrophage characterization from ischemic GC muscle in WT and *M*ɸ*Drp1*^KO^ mice following HLI. (**B**) Representative flow cytometry contour plots and quantification of numbers of F4/80^+^CD64^+^ macrophages isolated from ischemic GC muscles in WT and *M*ɸ*Drp1*^KO^ (MɸKO) mice on day 3 (D3) and D7 after HLI. (**C** and **D**) Immunofluorescence analysis of F4/80^+^ (green) and CD206^+^ (red) double-positive M2-like macrophages (**C**) or F4/80^+^ (green) and CD80^+^ (pink) double-positive M1-like macrophages (**D**) with DAPI (blue) in ischemic GC muscles in WT and MɸKO mice at indicated times after HLI. White arrowheads show indicated markers. Scale bars: 20 μm. Bottom panels show quantification (2-way ANOVA followed by Bonferroni’s multiple-comparison test). (**E**) Representative flow cytometry contour plots, histograms and (**F**) quantification of CD45^+^CD11b^+^F4/80^+^CD64^+^CD206^+^ M2-like macrophage and CD45^+^CD11b^+^F4/80^+^CD64^+^CD80^+^ M1-like macrophage numbers in ischemic GC muscles in WT and MɸKO mice at indicated times after HLI (2-way ANOVA followed by Bonferroni’s multiple-comparison test). Data are mean ± SEM. *n* = 4–6. *P < 0.05, **P < 0.01, ***P < 0.001. Note: CD80^+^CD206^+^ double-positive Mɸ in M1/M2 contour plots (**E**) were not included in the quantification shown in **F**.

Macrophages are required for revascularization during HLI (5–9). To determine whether myeloid DRP1 regulates macrophage accumulation and polarization in ischemic hind limbs (Figure 2A), we performed IF analysis. We observed an increase in F4/80^+^ macrophages in the ischemic muscles of WT mice on days 3 and 7 after HLI, which was reduced in *M*ɸ*Drp1*^KO^ mice (Supplemental Figure 2A). These findings were further confirmed by flow cytometry analysis, showing a reduction in CD45^+^CD11b^+^Ly6G^–^Ly6C^lo^ monocyte–derived F4/80^+^CD64^+^ macrophages isolated from the ischemic muscles of WT (*LysM*-Cre^+/–^ or *Drp1^fl/fl^*) and *M*ɸ*Drp1*^KO^ mice on days 3 and 7 after HLI (Figure 2B).

We next examined the polarization of F4/80^+^ macrophages into CD80^+^ proinflammatory M1-like macrophages and CD206^+^ antiinflammatory M2-like macrophages in ischemic muscles. The IF analysis demonstrated an increase in CD206^+^ M2-like macrophages and CD80^+^ M1-like macrophages in ischemic muscles of WT mice on days 3 and 7 after HLI. However, in *M*ɸ*Drp1*^KO^ mice, the number of CD206^+^ M2-like macrophages was reduced at both time points (Supplemental Figure 2B), while the number of CD80^+^ M1-like macrophages showed an increase on day 7 compared with WT mice (Supplemental Figure 2C). Colocalization analysis with F4/80^+^ cells revealed a decrease in F4/80^+^CD206^+^ M2-like macrophages in *M*ɸ*Drp1*^KO^ mice on days 3 and day 7 after HLI (Figure 2C). In contrast, F4/80^+^CD80^+^ M1-like macrophages were elevated in *M*ɸ*Drp1*^KO^ mice on day 7 after HLI compared with WT controls (Figure 2D). These findings were further confirmed by flow cytometry, which showed altered proportions of F4/80^+^CD64^+^CD206^+^ M2-like macrophages and F4/80^+^CD64^+^CD80^+^ M1-like macrophages in the ischemic muscles on days 3 and 7 after HLI (Figure 2, E and F), according to the gating strategy illustrated in Supplemental Figure 3. Notably, Ly6C^hi^-derived F4/80^+^CD64^+^ mature macrophages were negligible, with no detectable M1- and M2-like macrophages at these points. These results suggest that F4/80^+^CD64^+^ total macrophages originate from infiltrating CD45^+^CD11b^+^Ly6G^–^Ly6C^lo^ monocytes.

We next examined whether myeloid DRP1 deficiency–induced decrease in F4/80^+^ macrophages in ischemic muscles was due to a decrease in immune cell mobilization from the BM or their recruitment/infiltration or macrophage differentiation. To characterize the immune cells, we performed flow cytometry analysis in BM, peripheral blood, and ischemic muscle after HLI (gating strategy and antibody panel are shown in Supplemental Figure 3 and Table 2). We found that the numbers of Ly6G^+^ neutrophils and Ly6C^hi^ monocytes in BM (Supplemental Figure 4A) and peripheral blood (Supplemental Figure 4B) were similar between WT and *M*ɸ*Drp1*^KO^ mice on days 3 and 7 after HLI. Supplemental Figure 4C shows that, in ischemic muscles, the numbers of Ly6G^+^ neutrophils were increased in *M*ɸ*Drp1*^KO^ mice versus WT mice, while those of Ly6C^hi^ monocytes were similar between *M*ɸ*Drp1*^KO^ mice and WT mice on day 3 after HLI (Supplemental Figure 4C). Then, both Ly6G^+^ neutrophils and Ly6C^hi^ monocytes were reduced by day 7. These findings suggest that loss of myeloid DRP1 limits the polarization of antiinflammatory M2-like macrophages, which originate from CD45^+^CD11b^+^Ly6G^–^Ly6C^lo^ monocytes, as well as the differentiation of Ly6C^hi^ monocytes into mature F4/80^+^CD64^+^ macrophages. Importantly, this occurs without affecting the release of monocytes from the BM or their recruitment to the ischemic muscle. This in turn impairs ischemia-induced revascularization during HLI.

### Myeloid DRP1 deficiency increases proinflammatory genes and signaling and decreases antiinflammatory genes and signaling in ischemic muscle.

**Figure 3:**
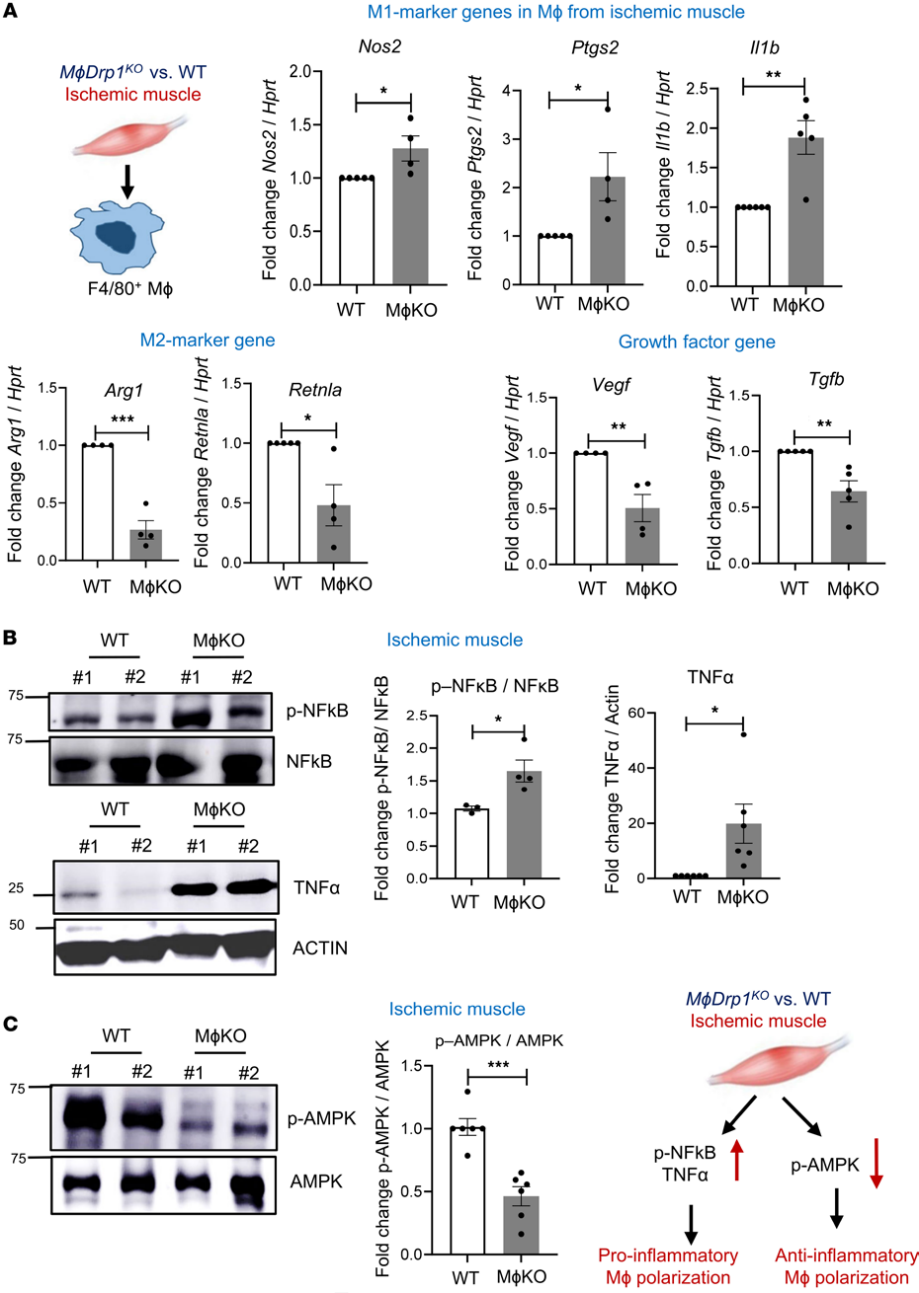
Myeloid *Drp1*^KO^ mice exhibited increase in proinflammatory genes and signaling and decrease in antiinflammatory genes and signaling in ischemic muscle. (**A**) Left: Schematic representation of magnetic bead–based F4/80^+^ macrophage purification from ischemic GC muscle on day 3 after HLI. These macrophages were used to analyze the proinflammatory gene (*Nos2*, *Ptgs2*, and *Il1b*), antiinflammatory gene (*Arg1* and *Retnla*), and proangiogenic growth factor gene (*Vegf* and *Tgfb*) mRNAs using qRT-PCR (fold increase relative to *Hprt*) in WT and *M*ɸ*Drp1*^KO^ (MɸKO) mice on day 3 after HLI (*n* = 4–5 mice per group, unpaired, 2-tailed Student’s *t* test). (**B** and **C**) Western blot analysis of proinflammatory markers (p-NF-κB and NF-κB), (TNF-α and actin [loading control]) (*n* = 3–6 mice per group) (**B**), and antiinflammatory markers (p-AMPK and AMPK) (*n* = 6 mice per group) (**C**), in ischemic tibialis anterior (TA) muscles of WT and MɸKO mice on day 3 after HLI. Blots for **B** and **C** were run separately on different gels using the same biological samples. The right panels show the quantification (unpaired, 2-tailed Student’s *t* test). Data are mean ± SEM. *P < 0.05, **P < 0.01, ***P < 0.001.

To confirm further the role of myeloid DRP1 in regulating macrophage polarization during HLI, we isolated F4/80^+^ macrophages from ischemic muscle on day 3 after HLI and found that proinflammatory M1-marker genes, including *Nos2*, *Ptgs2*, and *Il1b*, were upregulated. By contrast, antiinflammatory M2-marker genes, including *Arg1* and *Retnla*, were downregulated in *M*ɸ*Drp1*^KO^ mice compared with WT mice (Figures 3A). This was associated with a decrease in proangiogenic growth factor genes such as *Vegf* and *Tgfb* in macrophages isolated from ischemic muscles of *M*ɸ*Drp1*^KO^ mice versus WT mice (Figure 3A). Previous studies showed that proinflammatory NF-κB signaling and antiinflammatory AMPK signaling contribute to macrophage polarization toward M1 and M2 phenotypes, respectively (24–27). We found that expression of p-NF-κB, but not total NF-κB protein, was increased in ischemic muscles on day 3 after HLI of *M*ɸ*Drp1*^KO^ mice versus WT mice, which was associated with increased expression of proinflammatory TNF-α (Figure 3B). In contrast, expression of p-AMPK, but not total AMPK protein, was reduced in ischemic tissues on day 3 after HLI of *M*ɸ*Drp1*^KO^ mice versus WT mice (Figure 3C). Taken together, these results suggest that myeloid DRP1 deficiency enhanced the proinflammatory genes and p-NF-κB–mediated M1-like macrophage axis, while it decreased antiinflammatory genes and p-AMPK signaling associated with M2-like macrophage polarization in ischemic muscles. This imbalance resulted in impaired reparative neovascularization following HLI.

### Drp1^KO^ BMDMs under HSS exhibit mitochondrial hyperfusion, which is associated with enhanced M1 macrophage polarization and reduced M2 macrophage polarization.

**Figure 4:**
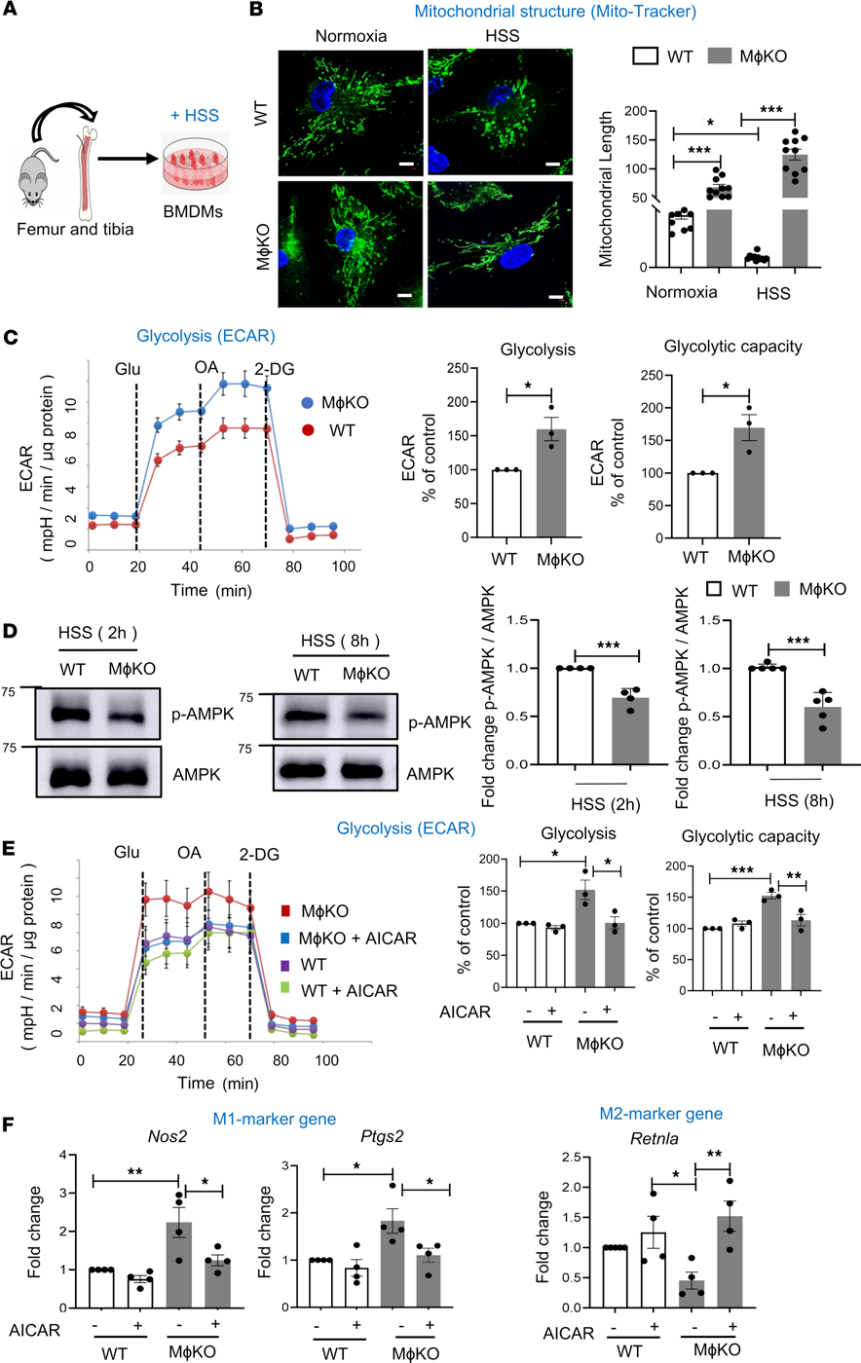
*Drp1*^KO^ BMDMs under HSS exhibited mitochondrial hyperfusion and hyperglycolysis, which was associated with decreased M2-like macrophages via reducing p-AMPK. (**A**) Schematic representation showing cultured mouse BMDMs exposed to hypoxia and serum starvation (HSS), an in vitro PAD model. BMDMs from WT and *M*ɸ*Drp1*^KO^ mice were cultured for 7 days and then exposed to HSS. (**B**) Analysis of mitochondrial structure by MitoTracker Green staining in WT and *M*ɸ*Drp1*^KO^ (MɸKO) BMDMs with or without HSS stimulation for 2 hours. Scale bars: 5 μm. Right panel shows quantification of mitochondrial length (*n* = number of cells analyzed from 3 independent experiments) by ImageJ (1-way ANOVA followed by Tukey’s multiple-comparison test). (**C**) Analysis of glycolysis measured by extracellular acidification rate (ECAR) using Seahorse XF analyzer in WT and MɸKO BMDMs under HSS for 2 hours. Right panels show quantification *n* = 3 (unpaired, 2-tailed Student’s *t* test). 2-DG, 2-deoxy-D-glucose; Glu, glucose; OA, oligomycin. (**D**) Western blot analysis for p-AMPK and AMPK expression in WT and MɸKO BMDMs with HSS stimulation for 2 hours and 8 hours. Right panels show quantification. *n* = 4–5 (unpaired, 2-tailed Student’s *t* test). (**E**) Effects of AMPK activator AICAR (pretreatment for 2 hours at 100 mM) on glycolysis (ECAR) measured by Seahorse XF analyzer (*n* = 3, 1-way ANOVA followed by Tukey’s multiple-comparison test). (**F**) mRNA expression for M1 markers (*Nos2* and *Ptgs2*) and M2 marker *Retnla* measured by qRT-PCR (*n* = 4, 1-way ANOVA followed by Tukey’s multiple-comparison test) in WT and MɸKO BMDMs under HSS for 2 hours (**E**) or 8 hours (**F**). Data are mean ± SEM. *P < 0.05, **P < 0.01, ***P < 0.001.

To explore the mechanisms by which DRP1 deficiency regulates macrophage polarization during ischemia, we performed in vitro studies using BMDMs isolated from WT and *M*ɸ*Drp1*^KO^ mice, subjected to HSS as an in vitro model for PAD (Figure 4A), as previously described (28, 29). MitoTracker Green staining revealed that WT BMDMs under HSS induced mitochondrial fission, peaking at 1 to 2 hours and returning to baseline levels by 8 hours (data not shown) (Supplemental Figure 5A). However, HSS did not alter p-Ser616-DRP1, p-Ser637-DRP1, DRP1 protein levels, or the expression of the mitochondrial fission protein MFF1 or fusion proteins MFN1 and OPA1 in BMDMs (Supplemental Figure 5B). In contrast, *Drp1*^KO^ BMDMs exhibited mitochondrial fusion 2 hours after HSS stimulation (Figure 4B). Electron microscopy further confirmed an increase in mitochondrial length and volume in *Drp1*^KO^ BMDMs following HSS stimulation compared with WT BMDMs (Supplemental Figure 5C).

We next investigated the role of DRP1 in macrophage polarization using BMDMs under in vitro HSS. Consistent with the in vivo HLI model, BMDM exposure to HSS increased M1 marker gene *Nos2* and decreased M2 marker gene *Retnla* in a time-dependent manner (Supplemental Figure 6A). *Drp1*^KO^ BMDMs with HSS exhibited enhanced M1-marker genes (*Nos2* and *Ptgs2*) and further decreased M2-marker genes (*Arg1* and *Retnla*) compared with *LysM-*Cre or *Drp1^fl/fl^* (WT) BMDMs with HSS (Supplemental Figure 6B). These findings suggest that loss of DRP1 in macrophages under HSS promotes proinflammatory macrophage polarization and reduces antiinflammatory macrophage polarization in vitro. Given that we observed a reduction in the total number of F4/80^+^ macrophages in ischemic muscles of *M*ɸ*Drp1*^KO^ mice, we further investigated whether DRP1 deficiency affects macrophage proliferation or cell death under HSS in vitro. There was no difference in cell death, as determined by the CCK-8 assay, or in cell proliferation, assessed using the BrdU assay, between WT and *Drp1*^KO^ BMDMs under HSS (Supplemental Figure 6, C and D).

To further explore whether DRP1 promotes the expression of typical M2-like macrophage markers, we performed bulk RNA sequencing (RNA-seq) on BMDMs isolated from *M*ɸ*Drp1*^KO^ and *LysM-*Cre^+/–^ *Drp1*^+/+^ (WT) mice following 8 hours of HSS stimulation (Supplemental Figure 7). The volcano plot revealed 2,325 differentially expressed genes, with 1,208 upregulated and 1,117 downregulated in *Drp1*^KO^ BMDMs compared with WT (Supplemental Figure 7A). Notably, similar to the gene expression profile observed in macrophages isolated from ischemic muscle, WT BMDMs exhibited a coordinated upregulation of M2-associated and proangiogenic genes, while *Drp1*^KO^ BMDMs showed increased expression of M1-associated genes and decreased proangiogenic gene expression (Supplemental Figure 7B). Gene Ontology (GO) (Supplemental Figure 7C) and Kyoto Encyclopedia of Genes and Genomes (KEGG) pathway (Supplemental Figure 7D) enrichment analyses confirmed that pathways associated with an M2-like phenotype were more active in WT BMDMs. The identified GO and KEGG pathways included genes involved in transcription, translation, cell activation, energy metabolism, intracellular transport, cell-cell interactions, and growth factors and cytokines linked to angiogenesis and muscle regeneration during ischemia (11, 30). These findings suggest that the loss of myeloid DRP1 impairs reparative M2 macrophage polarization under ischemic conditions.

### Drp1^KO^ BMDMs under HSS exhibit hyperglycolysis through reduced AMPK activation, leading to increased M1 macrophage and decreased M2 macrophage polarization.

Since proinflammatory macrophage polarization has been shown to undergo metabolic reprogramming from oxidative phosphorylation to aerobic glycolysis (12, 13), we measured the extracellular acidification rate (ECAR) using a Seahorse assay. Figure 4C shows that *Drp1*^KO^ versus WT BMDMs under HSS exhibited increased glycolysis and glycolytic capacity. To address the mechanism by which DRP1 deficiency in macrophages under HSS enhances glycolysis, we measured p-AMPK, which has been shown to promote antiinflammatory and proangiogenic M2-macrophage polarization via suppression of glycolysis (26, 27). In line with observations in ischemic tissue in vivo, we found a reduction in p-AMPK and its substrates, detected by antibodies targeting the p-AMPK substrate motif [LXRXX(pS/pT) in *Drp1*^KO^ BMDMs compared with WT BMDMs under HSS, but not under normoxia (Figure 4D and Supplemental Figure 8, B and C). To determine whether decreased AMPK activity contributes to enhanced glycolysis and proinflammatory macrophage polarization in *Drp1*^KO^ BMDMs under HSS, we performed rescue experiments using the AMPK activator 5-aminoimidazole-4-carboxiamide ribonucleotide (AICAR) (27, 31). We found that activating AMPK with AICAR, at a concentration that did not affect glycolysis in WT BMDMs under HSS, restored the reduced p-AMPK and its substrates while enhancing glycolytic activity in *Drp1*^KO^ BMDMs under HSS (Figure 4E and Supplemental Figure 8, B and C). Furthermore, this AMPK activation rescued the elevated expression of proinflammatory M1 genes *Nos2* and *Ptgs2* in *Drp1*^KO^ BMDMs under HSS, as compared with those observed in HSS-exposed WT BMDMs (Figure 4F). Notably, AICAR also rescued the reduced expression of genes associated with the antiinflammatory M2 phenotype in *Drp1*^KO^ BMDMs under HSS (Figure 4F). To address how DRP1 activates AMPK, we examined whether DRP1 regulates upstream AMPK kinases such as CAMKK and LKB1 in BMDMs exposed to HSS. We found a reduction in p-LKB1, but not in p-CAMKK, in HSS-stimulated *Drp1*^KO^ BMDMs compared with WT BMDMs (Supplemental Figure 8A). These findings suggest that DRP1 activates AMPK through LKB1 in macrophages under ischemic conditions.

### Drp1^KO^ BMDMs under HSS exhibit mitochondrial dysfunction and increased mitoROS production, leading to promoting M1-like macrophage polarization while inhibiting M2-like macrophage polarization.

**Figure 5:**
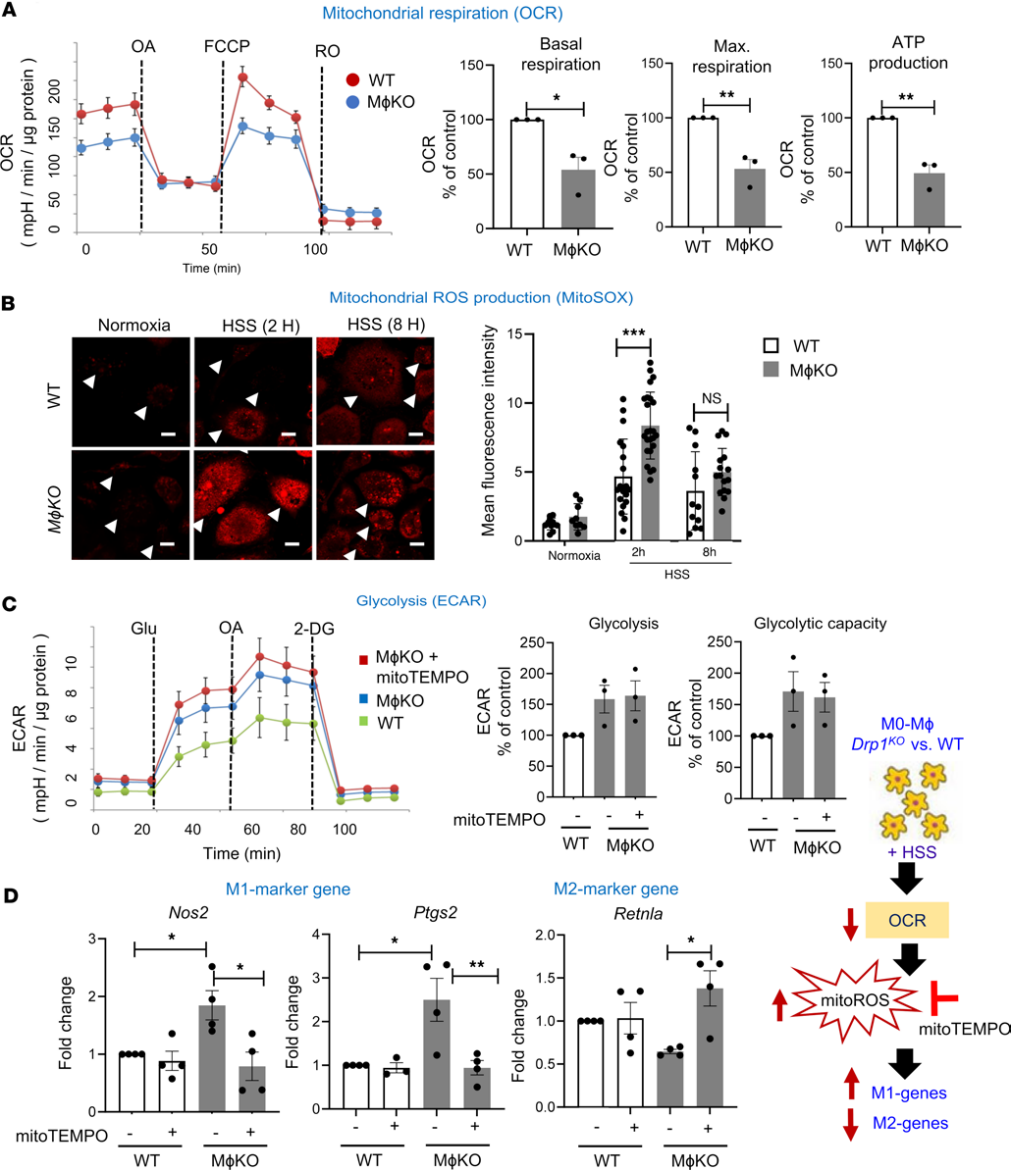
*Drp1*^KO^ BMDMs under HSS induced mitochondrial dysfunction and mitochondrial ROS (mitoROS) production, which in turn promoted M1-like macrophage polarization and reduced M2-like macrophage polarization. (**A**) Analysis of mitochondrial respiration measured by OCR using Seahorse XF analyzer in WT and *M*ɸ*Drp1*^KO^ (MɸKO) BMDMs under HSS for 2 hours. Right panels show quantification (*n* = 3, unpaired, 2-tailed Student’s *t* test). FCCP, carbonyl cyanide-*p*-trifluoromethoxyphenylhydrazone; OA, oligomycin; RO, rotenone. (**B**) mitoROS production measured by MitoSOX under normoxia or HSS for 2 hours and 8 hours in WT and MɸKO BMDMs. Scale bars: 5 μm. Right panels show the average fluorescence intensity quantified by ImageJ (*n* = number of cells analyzed from 3 independent experiments, 2-way ANOVA followed by Tukey’s multiple-comparison test). (**C** and **D**) Effects of Mito-TEMPO (pretreatment for 16 hours at 20 mM) on glycolysis (ECAR) using Seahorse assay (*n* = 3) (**C**) as well as mRNA expression for M1 markers (*Nos2* and *Ptgs2*) and M2 marker *Retnla* measured by qRT-PCR (**D**) (*n* = 3–4, 1-way ANOVA followed by Tukey’s multiple-comparison test) in WT and MɸKO BMDMs under HSS for 2 hours (**C**) or 8 hours (**D**). Data are mean ± SEM. *P < 0.05, **P < 0.01, ***P < 0.001.

Next, we examined the role of macrophage DRP1 in mitochondrial respiration by measuring OCR using the Seahorse assay. Figure 5A showed that *Drp1*^KO^ BMDMs under HSS exhibited a reduction in basal respiration, maximal respiration, and ATP production compared with WT BMDMs exposed to HSS. Note that basal OCR in both WT and *Drp1*^KO^ BMDMs under normoxic conditions was not different (Supplemental Figure 9A). This mitochondrial dysfunction was associated with excess mitoROS production in *Drp1*^KO^ BMDMs under HSS, as measured by MitoSOX fluorescence (Figure 5B). We then investigated the impact of increased mitoROS in *Drp1*^KO^ BMDMs on macrophage polarization under ischemic conditions. Our findings show that the mitochondria-specific O_2_^–^ scavenger, Mito-TEMPO, reversed the elevated expression of proinflammatory M1-genes, *Nos2* and *Ptgs2*, and reduced expression of the antiinflammatory M2-gene *Retnla*, in *Drp1*^KO^ BMDMs under HSS. Mito-TEMPO had no effects on M1 or M2 gene expression in WT BMDMs (Figure 5D) or on the elevated glycolysis (ECAR) in *Drp1*^KO^ BMDMs under HSS (Figure 5C). Notably, Mito-TEMPO restored the reduced basal respiration in *Drp1*^KO^ BMDMs, although it did not affect the decreased maximal respiration or ATP production of OCR, nor did it influence OCR in WT BMDMs (Supplemental Figure 9B). These results suggest that elevated mitoROS in *Drp1*^KO^ macrophages under HSS contributes to reduced basal respiration and M2-like polarization while promoting M1-like polarization, without altering glycolysis. Additionally, activation of AMPK by AICAR did not mitigate the excess mitoROS production in *Drp1*^KO^ BMDMs under HSS (Supplemental Figure 9C). Consequently, our results suggest that the concurrent elevation of excess mitoROS and glycolysis coupled with reduced AMPK activation collectively contribute to the promotion of M1-like macrophage polarization and the inhibition of M2-like macrophage polarization in *Drp1*^KO^ BMDMs under HSS conditions.

### Conditioned medium from WT BMDMs under HSS promotes angiogenesis in ECs, whereas medium from Drp1^KO^ BMDMs under HSS does not.

**Figure 6:**
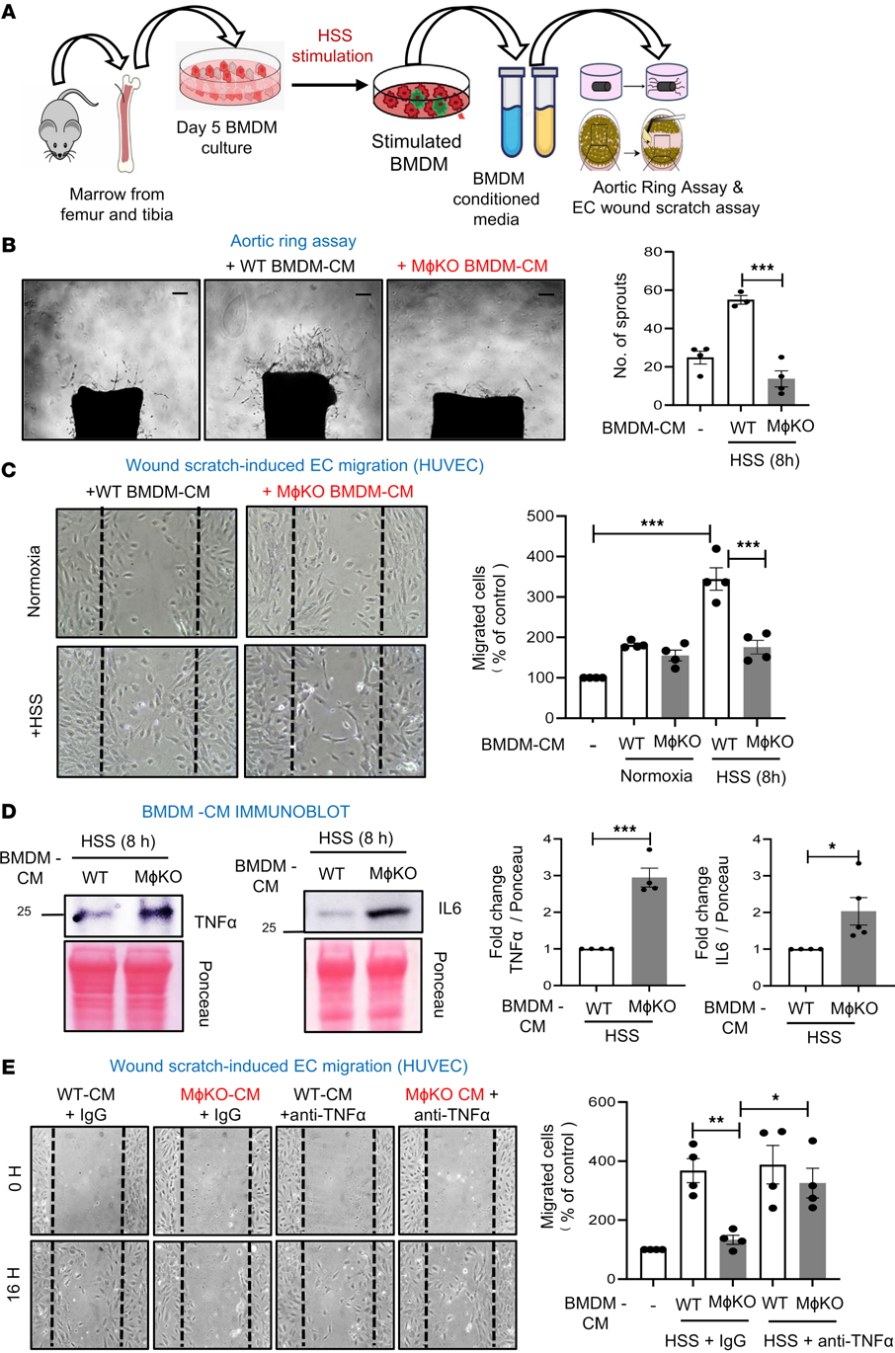
Conditioned medium (CM) from HSS-stimulated WT BMDMs, but not from *Drp1*^KO^ BMDMs, promoted angiogenesis in ECs. (**A**) Schematic representation of the isolation of CM from HSS-stimulated mouse BMDMs and its application on aortic ring assay and confluent human umbilical vein ECs (HUVECs) subjected to a scratch-wound assay. (**B**) Aortic ring assay showing the number of sprouts emerging from 1-mm aortic rings derived from WT mice, embedded on Matrigel, and incubated with CM (20 ng/mL) from HSS-stimulated WT or *M*ɸ*Drp1*^KO^ (MɸKO) BMDMs for 5 days. Scale bars: 1 mm (*n* = 4, 1-way ANOVA followed by Tukey’s multiple-comparison test). (**C**) EC migration using the scratch-wound assay on confluent HUVEC monolayers treated with CM from WT or MɸKO BMDMs exposed to normoxia or HSS for 8 hours. Representative bright-field images (left) and quantification of number of migrated cells per field (right) in HUVECs at 16 hours after wounding. Scale bars: 20 μm (*n* = 4, 1-way ANOVA followed by Tukey’s multiple-comparison test). (**D**) Representative protein expression of proinflammatory markers TNF-α and IL-6 and respective ponceau staining in CM from WT or MɸKO BMDMs stimulated with HSS for 8 hours. Blots were run separately on different gels using the same biological samples. Right panels show quantification (*n* = 4, unpaired, 2-tailed Student’s *t* test). (**E**) EC migration using the scratch-wound assay on confluent HUVEC monolayers treated with CM from WT or MɸKO BMDMs stimulated with HSS for 8 hours in the presence of either IgG (control) or anti–TNF-α antibody (1 μg/mL). Representative bright-field images (left) and quantification of number of migrated cells per field (right) for HUVECs at 0 hours and 16 hours after wounding. Scale bars: 20 μm (*n* = 4, 1-way ANOVA followed by Bonferroni’s multiple-comparison test). Data are mean ± SEM. *P < 0.05, **P < 0.01, ***P < 0.001.

To investigate how myeloid DRP1 affects angiogenesis in ECs, we analyzed the impact of paracrine factors from the conditioned media (CM) of WT and *Drp1*^KO^ BMDMs exposed to HSS using ex vivo aortic ring and scratch-wound assays (Figure 6A). CM from HSS-exposed WT-BMDMs, but not CM from HSS-exposed *Drp1*^KO^ BMDMs, increased the number of sprouts in the aortic ring assay (Figure 6B). Additionally, CM obtained from HSS-exposed WT-BMDMs, but not HSS-exposed *Drp1*^KO^ BMDMs, enhanced EC migration in a scratch-wound assay with confluent EC cultures (Figure 6C). In contrast, CM from normoxia-exposed WT or *Drp1*^KO^ BMDMs had a minimal effect on EC migration, with no difference between them (Figure 6C). The impaired proangiogenic effects of media from HSS-stimulated *Drp1*^KO^ BMDMs were associated with increased secretion of proinflammatory TNF-α and IL-6 (Figure 6D). Notably, the impaired EC migration was restored when the CM from HSS-stimulated *Drp1*^KO^ BMDMs were treated with an anti–TNF-α antibody, but not with a control IgG (Figure 6E). These results suggest that elevated TNF-α production by HSS-treated *Drp1*^KO^ macrophages contributes to reduced EC migration.

## Discussion

Macrophages play a vital role in restoring perfusion and promoting revascularization after ischemia, largely due to their ability to polarize into antiinflammatory M2 types and produce angiogenic factors. These functions are essential for the effective treatment of PAD (5–9). This study uncovers myeloid DRP1 as a key regulator of reparative macrophage responses during ischemia, with potential for therapeutic neovascularization via antiinflammatory macrophage polarization and metabolic reprogramming. Here we show that: (a) *M*ɸ*Drp1*^KO^ mice exhibited reduced limb perfusion, neovascularization, and muscle regeneration in ischemic muscle after HLI. These effects were accompanied by increased proinflammatory M1-like macrophage polarization, p-NF-κB and TNF-α levels, and decreased antiinflammatory M2-like polarization and p-AMPK in the ischemic muscle microenvironment. (b) In vitro, *Drp1*^KO^ BMDMs exposed to HSS, an in vitro model of PAD, showed enhanced glycolytic activity due to reduced p-AMPK, along with mitochondrial dysfunction and excess mitoROS production, leading to increased M1 and decreased M2 gene expression. (c) CM from HSS-treated *Drp1*^KO^ BMDMs showed elevated proinflammatory cytokine secretion and suppressed angiogenic responses in cultured ECs.

Growing evidence highlights the critical role of mitochondrial dynamics as a key intracellular signaling platform for regulating innate immune responses (17, 32, 33). However, the specific role of macrophage DRP1 in inflammatory responses remains a subject of conflicting findings. Some studies suggest that lack of DRP1 in macrophages and microglia reduces inflammatory responses (18, 21). Furthermore, in vivo pharmacological inhibition of DRP1 with Mdivi-1, which is known to have off-target effects, including inhibition of Complex I activity (34), reduces cytokine production and mitigates inflammation in infections and inflammatory diseases (20, 35, 36). Notably, the impact of myeloid DRP1 varies across different pathological conditions. For instance, macrophage DRP1 promotes vascular injury–induced intimal thickening by enhancing proinflammatory responses (18), whereas liver-specific DRP1 deficiency accelerates LPS-induced acute liver injury by increasing inflammation (19). In vitro studies using DRP1 knockdown in macrophages have shown increased mitochondrial fusion and IL-1β production via NLRP3 inflammasome activation (22). Therefore, the role of the mitochondrial fission GTPase DRP1 in modulating inflammatory phenotypes appears to depend on the context and cell type. Our current study provides the evidence to show the function of macrophage DRP1 in ischemia-induced neovascularization.

The present studies using myeloid *Drp1*–deficient mice with HLI, an experimental model of PAD, revealed that macrophage DRP1 plays a role in promoting reparative neovascularization in response to tissue ischemia. Flow cytometry and IF analyses found that *M*ɸ*Drp1^KO^* mice showed reduction in CD45^+^CD11b^+^Ly6G^–^Ly6C^lo^ alternative monocyte-derived naive M0-like F4/80^+^CD64^+^ macrophages and CD206^+^ M2-like macrophages on days 3 and 7 after HLI, without affecting Ly6C^hi^ monocyte recruitment into ischemic muscles. Notably, *M*ɸ*Drp1*^KO^ mice exhibited higher numbers of Ly6G^+^ neutrophils in the ischemic muscle on day 3 after HLI, followed by an increase in CD80^+^ M1-like macrophages and a decrease in M2-like macrophages by day 7. This aligns with previous studies of atherosclerotic plaques, where increased neutrophils promote the differentiation of infiltrating monocytes into M1 macrophages (37, 38). Furthermore, it has been shown that the enhanced clearance of infiltrated apoptotic neutrophils in ischemic muscles by macrophages contributes to the polarization of M2 macrophages, facilitating inflammation resolution and tissue regeneration (39). Notably, DRP1-mediated mitochondrial fission in macrophages is essential for the continuous clearance of apoptotic cells (40). Therefore, the elevated neutrophils in *M*ɸ*Drp1*^KO^ ischemic muscles may further limit M2 macrophage polarization and neovascularization. These findings suggest that, in ischemic conditions, macrophage DRP1 is critical for promoting monocyte-to-macrophage differentiation, enhancing antiinflammatory M2-like macrophage polarization and suppressing proinflammatory macrophage polarization. In this study, we also used BMDMs isolated from WT and *M*ɸ*Drp1*^KO^ mice with HSS stimulation, an in vitro model of PAD, and performed bulk RNA-seq, along with volcano plot analysis, and GO and KEGG pathway assessments. The results suggest that pathways associated with an M2-like phenotype and proangiogenesis were upregulated in WT BMDMs, while *Drp1*^–/–^ BMDMs exhibited increased expression of M1-related genes and decreased M2-related gene and proangiogenic gene expression.

Our study also found that the macrophage DRP1 deficiency–driven, impaired inflammatory M2-like macrophage phenotype in ischemic muscles is likely due to an increase in proinflammatory NF-κB signaling and a concurrent decrease in the activation of antiinflammatory p-AMPK. This finding aligns with previous reports demonstrating the detrimental role of proinflammatory NF-κB in hypoxia-induced ischemic brain injury and inflammation (24, 25). In contrast, AMPKa1 has been shown to promote HLI-induced arteriogenesis by regulating the production of angiogenic growth factors such as VEGF, TGF-β, and FGF2 by M2-like macrophages (26, 27). Note that proinflammatory macrophages shift their metabolic profile from oxidative phosphorylation to aerobic glycolysis (12, 13). AMPK has been shown to promote antiinflammatory and proangiogenic M2 macrophage polarization by suppressing proinflammatory glycolysis (26, 27). In line with our in vivo results in ischemic tissue, in vitro studies also demonstrated a reduction in AMPK activity, and enhanced induction of M1 genes *Nos2* and *Ptgs2* in *Drp1*^KO^ BMDMs under HSS when compared with WT BMDMs. Consequently, CM from *Drp1*^KO^ macrophages exposed to HSS showed increased secretion of proinflammatory cytokines TNF-α and IL-6, and exhibited impaired proangiogenic effects on cultured ECs compared with WT macrophages. Mechanistically, the reduced levels of p-AMPK and its substrate phosphorylation in *Drp1*^KO^ BMDMs were linked to enhanced glycolysis, as demonstrated by the ECAR. This effect was reversed with the AMPK activator AICAR. Additionally, AMPK activation decreased the elevated expression of M1 genes *Nos2* and *Ptgs2* in *Drp1*^KO^ BMDMs under ischemic conditions, while it increased the reduced expression of the M2 gene *Retnla*. We further investigated the mechanism by which DRP1 activates AMPK in macrophages under HSS. Our findings revealed that the upstream kinase of AMPK, p-LKB1, but not p-CAMKK, was reduced in *Drp1*^KO^ BMDMs following HSS stimulation. These results suggest that DRP1 senses HSS signals by increasing p-LKB levels, thereby activating AMPK in macrophages.

It is unclear how DRP1 is activated in BMDMs in response to HSS without phosphorylation. DRP1 activity is known to be regulated by various posttranslational modifications, such as phosphorylation, sumoylation, and *S*-nitrosylation at Cys644, among others (41, 42). This issue will be explored in more detail in future research. Additionally, the mechanism by which AMPK activation decreases glycolysis and proinflammatory macrophage polarization remains uncertain. It is reported that several downstream targets of AMPK suppress proinflammatory signaling in macrophages. These include the activation of PI3 kinase or SIRT1, leading to the deacetylation and inhibition of NF-κB activity (26, 43). Additionally, the antiinflammatory IL-10–mediated rapid activation of AMPK in macrophages is essential for the activation of the PI3K/Akt/mTORC1- and STAT3-mediated antiinflammatory polarization of macrophages (26, 44). AMPK also has also been shown to downregulate inflammatory metabolism by inhibiting HIF1α through the inactivation of mTORC1 (45). Further investigations are needed to elucidate the downstream targets of AMPK under ischemic conditions, which drive antiinflammatory signaling in macrophages.

**Figure 7:**
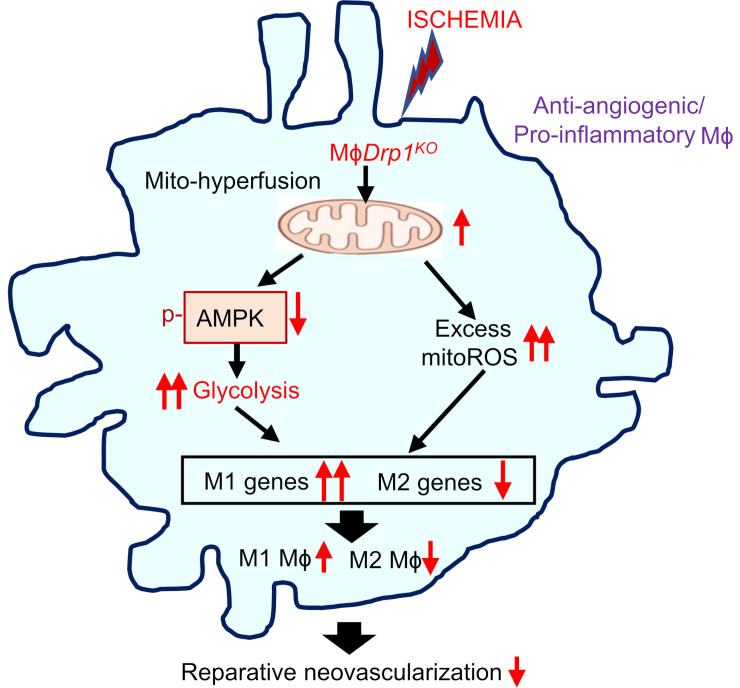
Schematic model illustrating that DRP1 deficiency in macrophages under ischemia induces metabolic reprogramming characterized by hyperglycolysis. This occurs due to reduced AMPK activation and a mitochondrial dysfunction–excess mitoROS axis, leading to an increase in proinflammatory M1-like macrophage polarization and a decrease in antiinflammatory M2-like macrophage polarization. Consequently, this impairs reparative neovascularization in response to ischemic injury.

Mitochondrial functions are crucial for cellular metabolism under ischemic conditions, and prolonged ischemia exacerbates mitochondrial dysfunction (16). While the role of macrophage DRP1 in driving proinflammatory responses under bacterial LPS stimulation is well documented (20, 36), its involvement in mitochondrial metabolism and inflammatory responses during ischemia remains unclear. For instance, after LPS stimulation, DRP1 activation in macrophages triggers excessive mitoROS production, which amplifies inflammatory cytokine release via NF-κB activation in a DRP1-dependent manner (20). In contrast, the present study demonstrates that *Drp1*^KO^ BMDMs exposed to HSS exhibited mitochondrial dysfunction, as evidenced by reduced OCR and increased mitoROS production. The mitoROS scavenger Mito-TEMPO restored the increased expression of M1 genes and decreased expression of M2 genes, but did not impact the elevated glycolysis or reduced maximal respiration and ATP production of OCR in *Drp1*^KO^ BMDMs. These findings suggest that elevated mitoROS induced by *Drp1*^KO^ macrophages under HSS contributes to enhancing proinflammatory macrophage polarization and suppressing antiinflammatory macrophage polarization without affecting glycolysis or OCR. It has been reported that AMPK and mitoROS can regulate each other (46, 47). For example, AMPK-mediated DRP1 regulation prevents EC dysfunction by suppressing mitoROS and ER stress (47). Under hypoxic conditions, mitoROS can directly activate AMPK through phosphorylation, independently of the AMP/ATP ratio (46). However, in the current study, AICAR treatment did not reduce the elevated mitoROS in *Drp1*^KO^ BMDMs under HSS. Thus, these findings suggest that in *Drp1*^KO^ BMDMs exposed to HSS, hyperglycolysis is driven by the inhibition of AMPK activation independently of mitoROS, while excess mitoROS production occurs in parallel. These mechanisms may contribute to impaired neovascularization with enhanced M1 and reduced M2 macrophage polarization in *M*ɸ*Drp1*^KO^ mice (Figure 7).

To explore the role of myeloid DRP1 in angiogenesis of ECs, we examined the effects of paracrine factors from the CM of WT and *Drp1*^KO^ BMDMs subjected to HSS on angiogenic responses. We utilized an ex vivo aortic ring assay and a scratch-wound-induced EC migration assay for this purpose. Our results indicated that CM obtained from HSS-treated *Drp1*^KO^ BMDMs exhibited reduced proangiogenic function compared with that from HSS-treated WT BMDMs, which was associated with increased secretion of proinflammatory cytokines, including TNF-α and IL-6. Notably, the impaired migration of ECs was restored when the CM from HSS-stimulated *Drp1*^KO^ BMDMs was treated with an anti–TNF-α antibody. These findings indicate that proinflammatory cytokines, particularly TNF-α released from HSS-stimulated *Drp1*^KO^ macrophages, contributes to impairing angiogenesis in ECs. This is in line with our in vivo observations and underscores the crucial role of macrophage DRP1 in facilitating communication between macrophages and ECs, which is necessary for reparative neovascularization following ischemia.

In summary, our findings underscore the essential role of macrophage DRP1-mediated mitochondrial dynamics in driving metabolic reprogramming toward antiinflammatory macrophages, thereby enhancing neovascularization in response to ischemia. Specially, we have identified the activation of AMPK and the concurrent reduction in excessive mitoROS production as crucial downstream targets through which myeloid DRP1 orchestrates the metabolic reprogramming and mitochondrial functions of macrophages, fostering enhanced neovascularization and tissue repair in response to ischemic injury. Future studies will focus on exploring the role of endothelial DRP1 in ischemia-induced neovascularization (48). Furthermore, given the encouraging outcomes from clinical trials using intramuscular cell therapy with BM mononuclear cells in patients with critical limb ischemia (4), our findings suggest that targeting macrophage DRP1 could be a promising therapeutic approach for treating PAD.

## Methods

### Sex as a biological variable.

All experiments involving animals were conducted using both male and female mice at 8 to 12 weeks of age. No phenotype differences were observed between male and female mice in this study.

### Animal study.

Room temperature and humidity were maintained at 22.5°C and between 50% and 60%, respectively. All mice were maintained under a 12-hour light/12-hour dark cycle in individually ventilated cages, with a maximum of 5 or a minimum of 2 mice per cage. *M*ɸ*Drp1*^KO^ mice were generated by crossing *Drp1^fl/fl^* mice (provided by Hiromi Sesaki, Johns Hopkins University, Baltimore, Maryland, USA) with *LysM*-Cre^Tg/Tg^ mice (JAX stock 004781 on a C57BL/6J background). Male mice heterozygous for the *Drp1*-floxed allele and *LysM-*Cre transgene (*Drp1^fl/+^* *LysM*-Cre^Tg/+^) were cross-bred with homozygous *Drp1*-floxed female mice. The *LysM-*Cre^+/–^ *Drp1^fl/fl^* (*M*ɸ*Drp1*^KO^) genotype, as well as their littermate controls (*LysM-*Cre^+/–^ *Drp1*^+/+^ or *LysM-*Cre^–/–^ *Drp1^fl/fl^*), were used for experiments.

### HLI model.

Mice were subjected to unilateral hindlimb surgery under anesthesia with intraperitoneal administration of ketamine (87 mg/kg) and xylazine (13 mg/kg). We performed ligation and segmental resection of the left femoral artery. Briefly, the left femoral artery was exposed, ligated both proximally and distally using 6-0 silk sutures, and the vessels between the ligatures were excised without damaging the femoral nerve. Skin closure was done using 6-0 nylon sutures. We measured ischemic (left)/non-ischemic (right) limb blood flow ratio using a laser Doppler blood flow (LDBF) analyzer (PeriScan PIM 3 System, Perimed), as we reported previously (49, 50). Mice were anesthetized and placed on a heating plate at 37°C for 10 minutes to minimize temperature variation. Before and after surgery, LDBF analysis was performed in the plantar sole. Blood flow was displayed as changes in the laser frequency, represented by different color pixels, and mean LDBF values were expressed as the ratio of ischemic to non-ischemic LDBF.

### Histological and Western blot analyses.

See Tables 1 and 2. For cryosections, mice were euthanized and perfused through the left ventricle with saline, limbs were fixed in 4% paraformaldehyde (PFA) overnight and incubated with 30% sucrose, and GC muscles were embedded in OCT compound (Sakura Finetek). Cryosections (7 μm) for capillary density were stained with anti–mouse CD31 antibody (MEC 13.3, BD Bioscience). For immunohistochemistry, we used R.T.U. Vectorstain Elite (Vector Laboratories) followed by DAB visualization (Vector Laboratories). Arterioles were stained with a Cy3-conjugated anti-αSMA antibody (1A4, Sigma-Aldrich). Macrophages were labeled with anti-F4/80 (BM8, BioLegend), and M1 macrophage and M2 macrophages were labeled with anti-CD80 (16-10A1, BioLegend) and anti-CD206 (C068C2, BioLegend) antibodies, respectively (Table 1). Images were captured by Keyence microscope (BZ-X800) or confocal microscopy (Zeiss) and analyzed by ImageJ v1.52 (NIH) or LSM510 software (Zeiss), respectively. Western blot analysis was performed as previously described (23) (Table 2).

### Isolation and primary culture of BMDMs.

BM cells were harvested from hind leg tibiae and femurs from 2 WT and *M*ɸ*Drp1*^KO^ mice and pooled. Briefly, BM cells were flushed from the bone with a 27-G needle in Dulbecco’s PBS (DPBS) and filtered using a 70 μm filter. Cells were cultured and differentiated in DMEM (Gibco) supplemented with antibiotics, 10% FBS, and 20% CM from the L929 cell line (enriched in M-CSF) for 7 days in polystyrene culture plates, and nonadherent cells were washed and removed every alternate day. The resulting BMDM population was determined by staining with anti-CD11b, anti-Ly6C, and anti-F4/80 antibodies and assessed by flow cytometry (Table 3).

### In vitro HSS.

Cultured BMDMs were washed twice to remove traces of FCS and then incubated in starvation medium (209-250, Cell Applications Inc.) and subjected to hypoxia (2% O_2_) for indicated times (28, 29).

### CM preparation from HSS-stimulated BMDMs.

Cultured BMDMs were exposed to HSS for 8 hours, and CM was collected, filtered through 0.2 μm filters, and stored at –80°C with protease and peptidase inhibitor (1861280, Thermo Fisher Scientific) until further use. Prior to the experiment, BMDM CM was thawed and concentrated using 3K Amicon ultra centrifugal tubes (UFC800396). Briefly, the CM was centrifuged at 3846*g* for 8 minutes at 4°C. Total protein concentration was determined using the Bradford assay.

### BMDM mitochondrial structure and mitoROS measurement.

Normoxia-exposed or HSS-stimulated BMDMs with or without mito-TEMPO (ALX-430-150-M005, Enzo) treatment were stained with MitoTracker Green FM (M7514, Invitrogen) or mitoSOX Red mitochondrial superoxide indicator (M36008, Invitrogen) to visualize mitochondrial structure or mitoROS, respectively. Images were acquired by confocal microscopy (Zeiss LSM 710).

### FACS analysis.

See Table 3. Mouse whole blood and BM cells were collected by cardiac puncture and from femurs and tibias, respectively, using a 27-G needle and EDTA-coated 1 mL syringes and placed in 1.5 mL EDTA-coated Eppendorf tubes for 30 minutes at room temperature. The peripheral blood was spun down at 500*g* for 1 minute at 4°C. The supernatant was discarded, and the cells were resuspended in 1 mL of RBC lysis buffer (00-4300-54, Invitrogen) for 10 minutes at room temperature in the dark. Samples were then washed with DPBS with 2% FCS and centrifuged at 500*g* for 5 minutes at 4°C. Cells were resuspended in cold DPBS with 2% FCS and placed on ice. Excised GC muscles were minced and enzymatically digested in DPBS containing 1 mg/mL collagenase type I (C0130, Sigma-Aldrich), 18 mg/mL collagenase type XI (C7657, Sigma-Aldrich), 1 mg/mL hyaluronidase (H3506, Sigma-Aldrich), and 50 U/mL DNase (D4263, Sigma-Aldrich) at 37°C for 1 hour. Cells were spun and resusupended in DPBS with 2% FCS, followed by passing through 70 μm and 40 μm filters and counted. The cell suspension was incubated with blocking buffer consisting of anti–mouse CD16/CD32 (14-0161-85, eBioscience) and 2% FCS for 15 minutes in ice. Cells were stained with fluorophore-labeled specific antibodies for CD45, CD11b, Ly6G, Ly6C, F4/80, CD64, CD206, and CD80, and then incubated with a fixable viability dye for live cell gating (65-0866-14, Invitrogen) at 4°C for 30 minutes. Simultaneously, fluorophore-labeled isotype controls were used to exclude false positives due to nonspecific antibody binding. All antibodies were diluted at 1:100. Unstained cells and compensation beads (01-3333-42, Invitrogen) served as single-color controls for autocompensation. After staining, cells were washed, fixed with 4% PFA, and analyzed using an Attune Nxt flow cytometer (A24858, Thermo Fisher Scientific). The recorded data were processed using FlowJO 10.10 software (BD Biosciences).

### Isolation of macrophages from GC muscle.

The GC muscles were minced and enzymatically digested as described above in *FACS analysis*. Cells were then magnetically labeled with an anti-F4/80 Microbeads (130-110-443, Miltenyi Biotec) and isolated through magnetic sorting (Miltenyi Biotec).

### Scratch-wound cell migration assay.

Confluent ECs, following 12 hours of serum starvation (0.1% serum media), were scraped using sterilized 10-μL pipette tips and then washed with 0.1% serum media (23). The ECs were incubated with CM (20 ng/mL) derived from HSS-treated WT or *Drp1^–/–^* BMDMs 16 hours with an anti–TNF-α antibody or IgG vehicle control. Images were captured immediately at 0 hours and 16 hours after the wounding.

### Ex vivo aortic ring assay.

Aortic rings from WT mice (1 mm in size) were prepared and placed in 48-well plates coated with growth factor–reduced Matrigel (Corning). The aortic rings were cultured for 5 days, with treatment every other day using CM (20 ng/mL) derived from HSS-treated WT or *Drp1^–/–^* BMDMs. Branch points or sprouting of aortic rings were quantified using ImageJ software.

### RNA-seq.

BMDMs isolated from *M*ɸ*Drp1*^KO^ or *LysM*-Cre^+/–^ *Drp1*^+/+^ (WT) mice simulated with HSS for 8 hours were used. Total RNA was extracted using the RNeasy kit (Qiagen), and RNAs were quantified by spectrophotometry (ND-2000, NanoDrop, Thermo Fisher Scientific). The quality of RNAs was determined using electrophoresis (2100 Bioanalyzer, Agilent Technologies) prior to next-generation sequencing in the Genome Technology Access Center (GTAC) at Washington University. Samples with RNA integrity number (RIN) values greater than 8.0 were subjected to polyA selection, chemical fragmentation, random hexamer priming, cDNA synthesis, and adapter ligation using the TruSeq RNA Library Prep Kit (Illumina), followed by paired-end multiplexed sequencing (HiSeq 2500, Illumina) according to the manufacturer’s instructions.

### RNA-seq analysis.

The FASTQ RNA-seq read files were quantified using Kallisto (v0.46.1, California Institute of Technology) with the transcriptome indices built on the cDNA FASTA files from Ensembl mouse genome assembly GRCm39. Genes with lower than 10 total read counts were filtered for subsequent differential expression analysis. Differential expression analysis was performed using R package DESeq2 (v1.32.0, The University of North Carolina at Chapel Hill) by comparing *Drp1*^KO^ and WT BMDM samples, and the differentially expressed genes were identified using fold change greater than 1.25 and *P* less than 0.05. The GO Consortium and KEGG pathway databases were used to perform GO and pathway enrichment analysis with the R package clusterProfiler (v4.0.2, Southern Medical University).

### Quantitative RT-PCR.

Total RNA was prepared from cells or tissues using Tri Reagent (Molecular Research Center Inc.). and phenol/chloroform. Reverse transcription (RT) was carried out using a high-capacity cDNA reverse transcription kit (Applied Biosystems) with 2 μg of total RNA. PCR was performed as per manufacturer’s protocol using the ABI Prism 7000 Sequence Detection System 26 (Applied Biosystems) and the QuantiFast SYBR Green PCR kit (Qiagen) for specific genes. Primer sequences for qRT-PCR are listed in Table 4. The expression of genes was normalized and expressed as fold change relative to *Hprt*.

### Statistics.

Each experiment was repeated at least 3 times and data are presented as mean ± SEM. Comparison between 2 groups were analyzed by unpaired, 2-tailed Student’s *t* test. Experiments with more than 2 subgroups were analyzed by ANOVA followed by the Tukey’s post hoc or Bonferroni’s multiple-comparison analysis to specify the significance between group differences. A *P* value of less than 0.05 was considered statically significant. Statistical tests were performed using Prism v10 (GraphPad Software).

### Study approval.

All animal studies were carried out following protocols approved by the Institutional Animal Care Committee and institutional Biosafety Committee at Augusta University.

### Data availability.

Gene expression data are available under NCBI GEO accession number GSE280186. All raw data associated with findings presented in the manuscript are included in the Supporting Data Values file.

## Author contributions

MUF, TF, and SY designed the study. SY, VS, AD, DA, SN, and SK performed/assisted research. MUF, TF, SY, VO, and YS analyzed data. YS and VCG discussed data and provided inputs. MM and SK performed mouse genotyping. VCG provided materials. MUF, TF, and SY wrote the manuscript.

## Supplementary Material

Supplemental data

Unedited blot and gel images

Supporting data values

## Figures and Tables

**Table 1:**
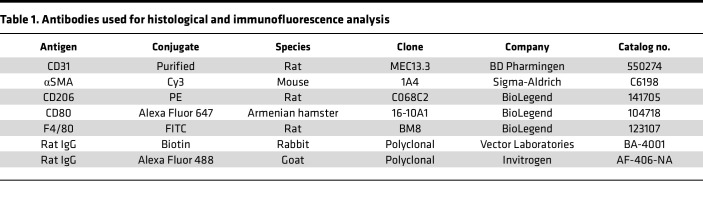
Antibodies used for histological and immunofluorescence analysis

**Table 2:**
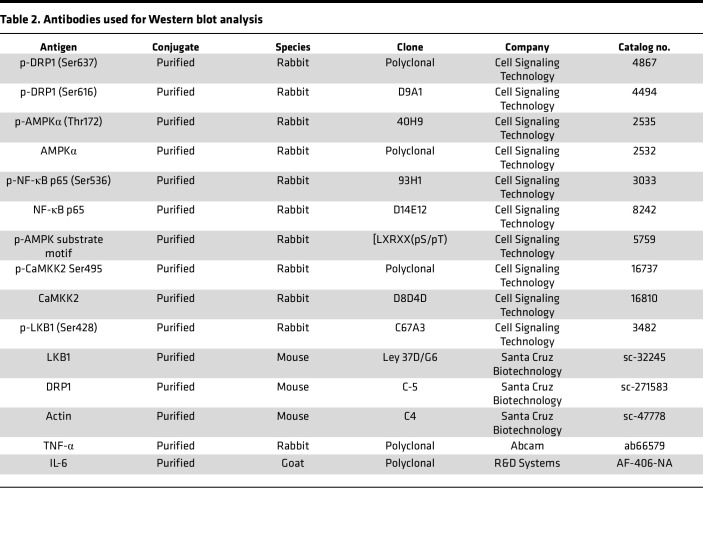
Antibodies used for Western blot analysis

**Table 3:**
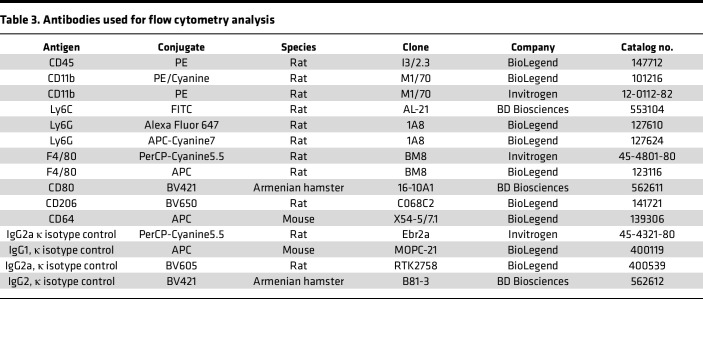
Antibodies used for flow cytometry analysis

**Table 4:**
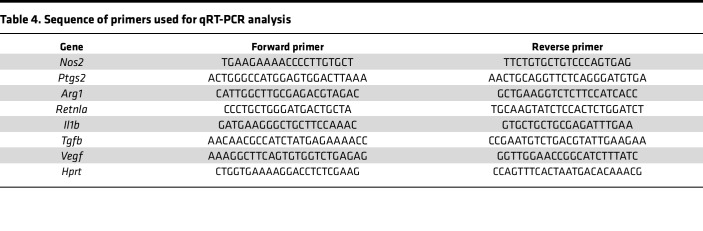
Sequence of primers used for qRT-PCR analysis

## Version 1 11/26/2024

In-Press Preview

## Version 2 01/09/2025

Electronic publication
