# Supplementary material for: Myeloid DRP1 deficiency limits revascularization in ischemic muscles via inflammatory macrophage polarization and metabolic reprogramming
Source: JCI Insight. 2025 Jan 9;10(1):e177334. doi: 10.1172/jci.insight.177334 (PMC11721294; doi:10.1172/jci.insight.177334)
Supplement: Supplemental data [file jciinsight-10-177334-s092.pdf]

Supplemental Material

Myeloid DRP1 Deficiency Limits Revascularization in Ischemic Muscles via Inflammatory Macrophage Polarization and Metabolic Reprograming

Shikha Yadav[1], Vijay Ganta[1,2], Sudhahar Varadarajan[1,3,4], Vy Ong[5], Yang Shi[5], Archita Das[1,3,4], Dipankar Ash[1,3], Sheela Nagarkoti[1], Margorzata McMenamin[1,4], Stephanie Kelley[1], Tohru Fukai[1,3,4], Masuko Ushio-Fukai[1,2]

[1]Vascular Biology Center, [2]Department of Medicine (Cardiology), [3]Department of Pharmacology and Toxicology, Medical College of Georgia at Augusta University,
[4]Charlie Norwood Veterans Affairs Medical Center, Augusta GA 30912
[5]Biostatistics and Bioinformatics Core, Karmanos Cancer Institute, Department of Oncology, Wayne State University School of Medicine, Detroit, MI 48201.

Running Title: Macrophage Drp1 and angiogenesis

***Address correspondence to**:
Masuko Ushio-Fukai, Ph.D
Vascular Biology Center
Department of Medicine (Cardiology)
Medical College of Georgia at Augusta University
1460 Laney-Walker Blvd, CB-3212A
Augusta, GA 30912, USA
Email: mfukai@augusta.edu
ORCID iD https://orcid.org/0000-0001-7048-2381

Supplemental Figure S1

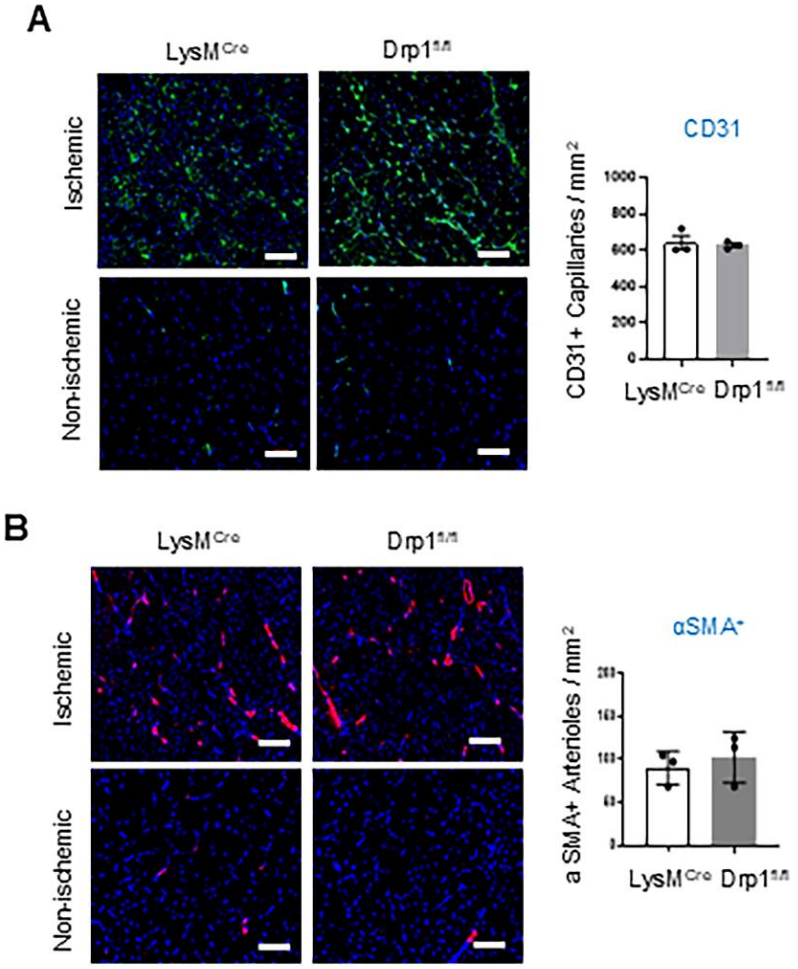

Figure S1: *Drp1*$^{fl/fl}$ and *LysM*-Cre mice exhibited no difference in the number of CD31^{+} capillary endothelial cell (EC) and αSMA^{+} arterioles in ischemic muscles following hindlimb ischemia (HLI). A and B. Immunofluorescence (IF) images (left) and quantification (right) of CD31^{+} capillary-like EC (A) and α-smooth muscle cells (SMA^{+}) (arterioles) (B) in ischemic and non-ischemic GC muscles at day 21 post-HLI. scale bar=20 µm. n=3. Data are mean ± SEM.

Supplemental Figure S2

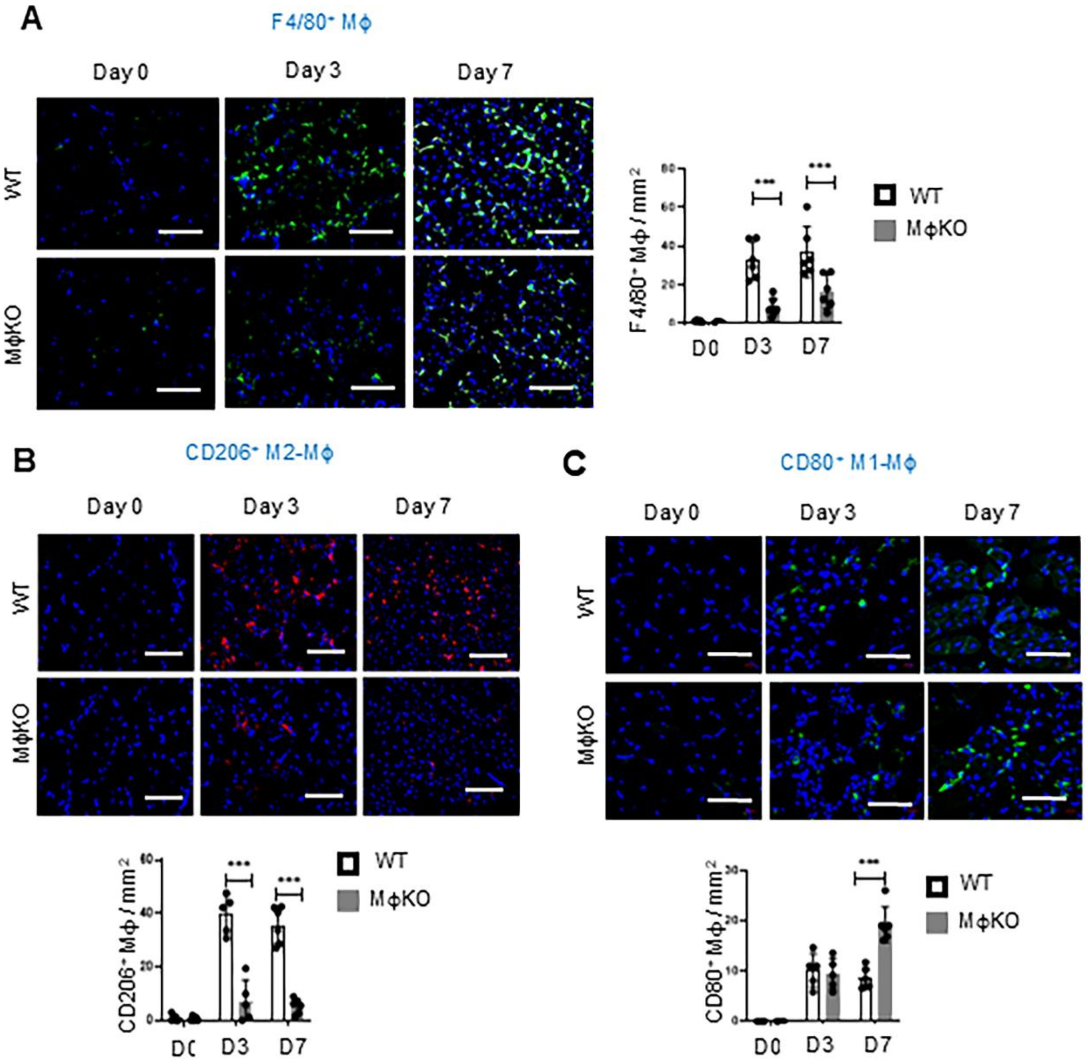

Figure S2: Myeloid *Drp1*^{KO} mice exhibited a reduction in F4/80^{+} macrophages and anti-inflammatory CD206^{+} M2-like macrophages, along with an increase in pro-inflammatory CD80^{+} M1-like macrophages in ischemic muscles. A. IF analysis of F4/80^{+} macrophages (green) with DAPI (blue) staining in non-ischemic and ischemic GC muscles of WT and MϕKO mice at the indicated times after HLI. Scale bar=20µm. **B-C** IF analysis of CD206^{+} (red) M2-like macrophages (**B**) and CD80^{+} (green) M1-like macrophages (**C**) with DAPI (blue) in ischemic GC muscles of WT and MϕKO mice at the indicated times post-HLI. Scale bar=20µm, bottom panels show quantification. n=3-5 mice per group, (two-way ANOVA followed by Tukey's multiple comparison test). Data are mean ± SEM. ***p<0.001.

Supplemental Figure S3

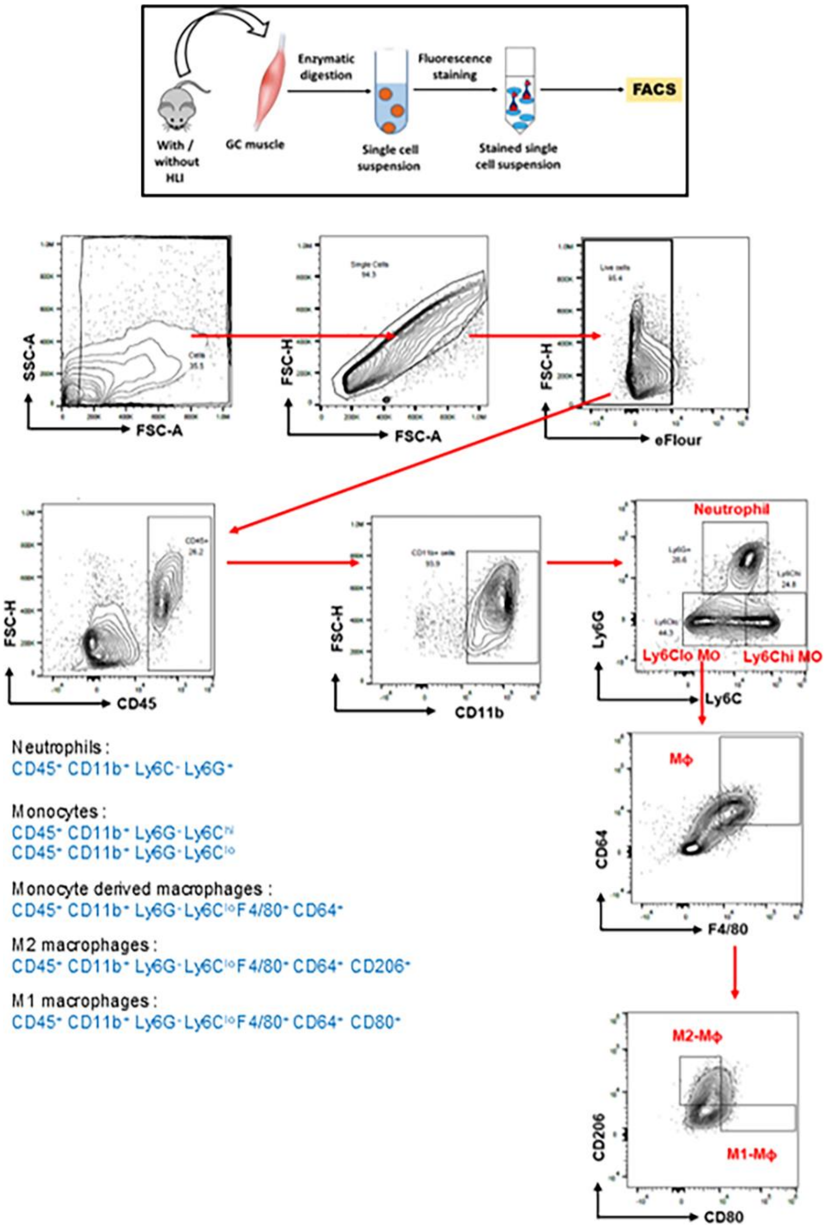

Figure S3: Schematic representation of the gating strategy for flowcytometry based immunophenotyping of gastrocnemius muscle. Gating strategy for flow cytometry-based analysis to identify and quantify neutrophils, monocytes, naïve (M0) macrophages, pro-inflammatory M1 macrophages, and anti-inflammatory M2 macrophages isolated from ischemic GC muscle at the indicated time after HLI. Cells were first gated based on FSC-A and SSC-A for singlets, followed by gating for live cells and $CD45^{+}/CD11b^{+}$ double-positive leukocytes. Neutrophils ($Ly6G^{+}$), monocyte ($Ly6C^{hi}$), naïve M0 Macrophages ($Ly6C^{lo}/F4/80^{+}/CD64^{+}$), M1 macrophages ($Ly6C^{lo}/F4/80^{+}/CD64^{+}/CD80^{+}$), and M2 macrophages ($Ly6C^{lo}/F4/80^{+}/CD64^{+}$ $/CD206^{+}$) were quantified. Note $CD80^{+}CD206^{+}$ double positive Mϕ in M1/M2 contour plots are not considered in the quantification.

Supplemental Figure S4

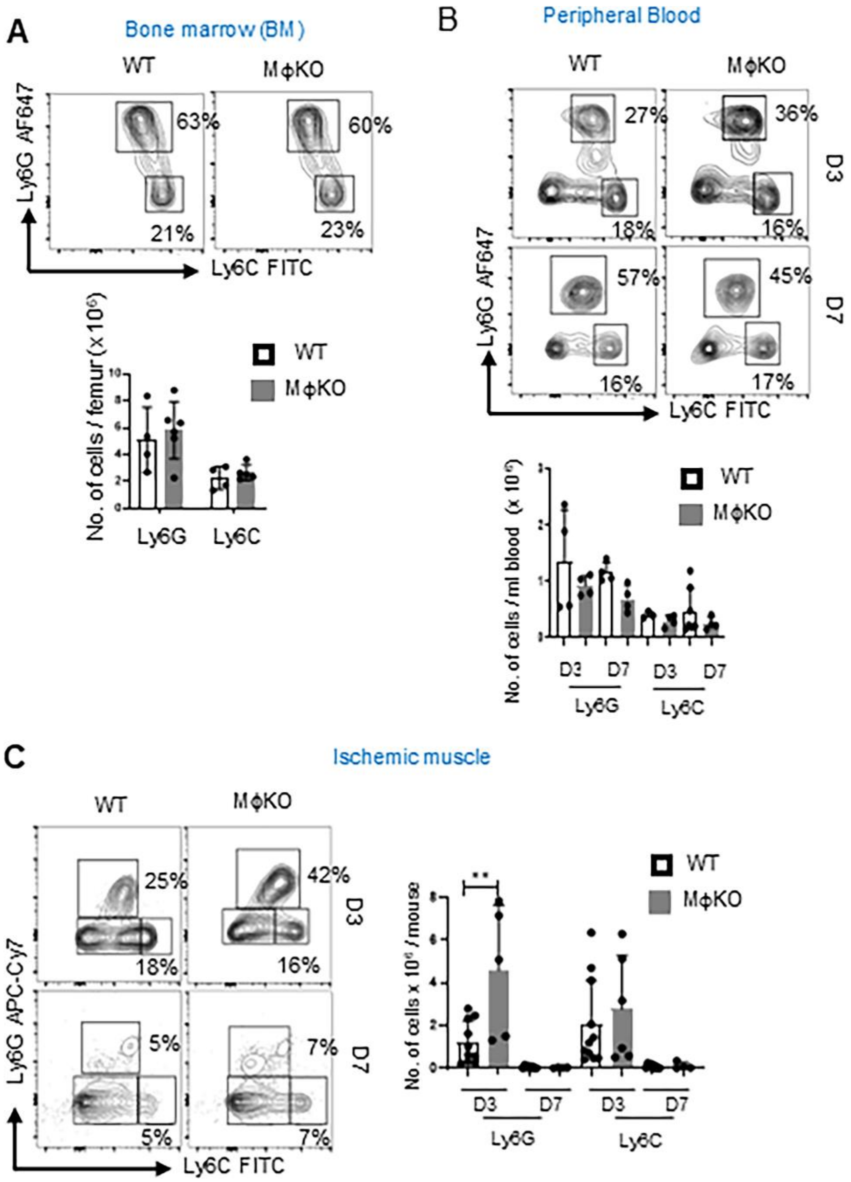

Figure S4: Neutrophils and monocytes in peripheral blood, bone marrow (BM), and ischemic GC muscles of WT and Myeloid *Drp1*^{KO} mice following HLI. Representative flow cytometry contour plots and quantification of Ly6G^{hi} neutrophils and Ly6C^{hi} monocytes in the BM (A) and peripheral blood (B) at day 3 post-HLI, as well as in ischemic GC muscle at days 3 and 7 post-HLI in WT and MϕKO mice. n=3-10 mice per group. Data are mean ± SEM. n=3-5. **p<0.01.

Supplemental Figure S5

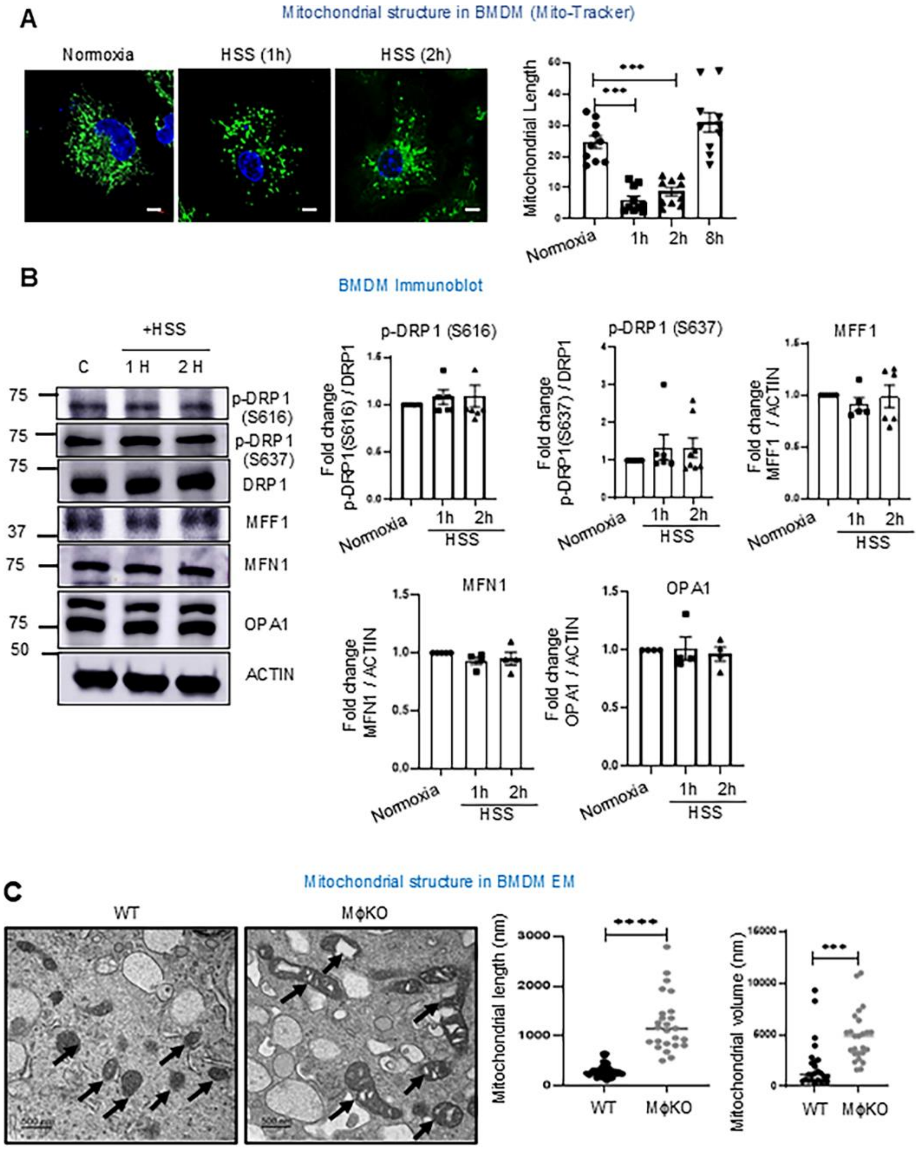

Figure S5: BMDMs under HSS exhibited increased mitochondrial fission without changes in the expression of key mitochondrial fission/ fusion proteins, while *Drp1*^{KO} BMDMs under HSS showed mitochondrial fusion. **A.** Mitochondrial structure in WT-BMDMs stained with MitoTracker Green after HSS stimulation for the indicated time (left). The right panel shows quantification of mitochondrial length measured using image J (right). Scale bar=5μm, n=number of cells analyzed from 3 independent exp. ***p<0.001 (one-way ANOVA followed by Tukey's multiple comparison test). **B.** Western blot analysis of p-DRP1-S616, p-DRP1-S637, total DRP1, MFF1, MFN1, OPA1 and ACTIN (loading control) in WT-BMDM exposed to HSS for the indicated time. The right panels show quantification. Data are mean ± SEM. n=4-5. **C.** Electron microscopy images and quantification of mitochondrial length and volume in WT and MϕKO BMDMs after 1 h of HSS stimulation. n=number of cells analyzed per group. Data are mean ± SEM. ****p<0.0001 (unpaired t test).

Supplemental Figure S6

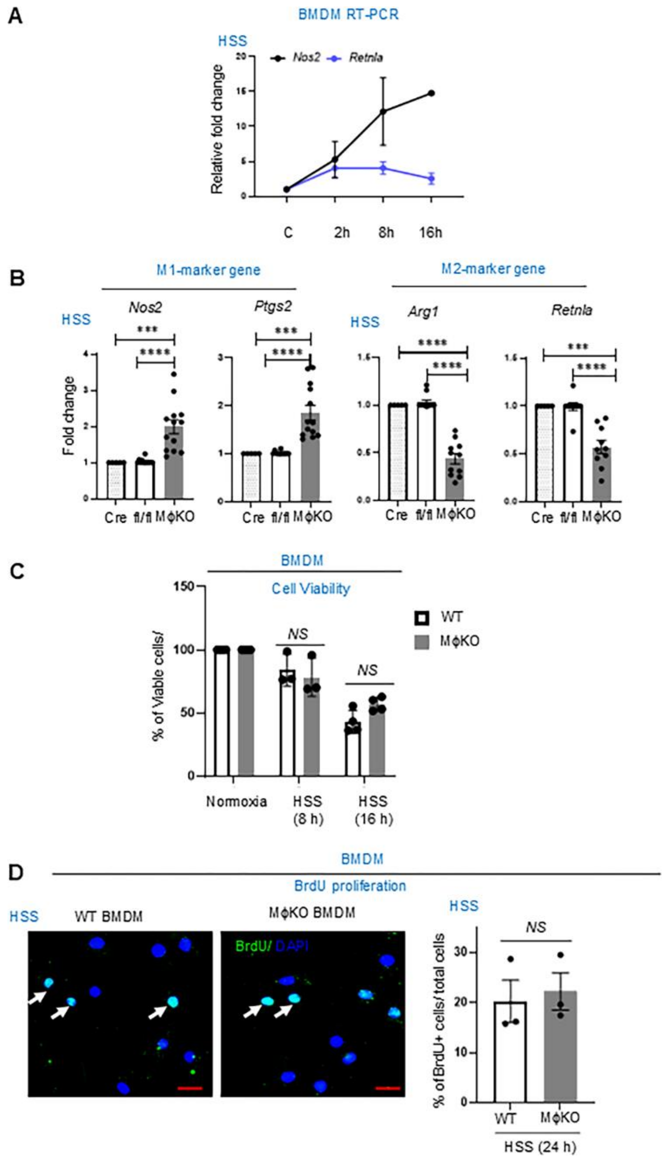

Figure S6: *Drp1*^{KO} BMDMs under HSS showed an increase in M1-marker genes and a decrease in M2-marker gene expression without affecting cell viability or proliferation. **A.** Quantitative RT-PCR (relative to *Hprt*) analysis of M1 marker gene *Nos2* and M2 marker gene *Retnla* mRNA levels in WT-BMDMs exposed to HSS for the indicated time. Data are expressed as fold change from normoxic controls. **B.** mRNA levels of M1 markers (*Nos2* and *Ptgs2)* and M2 markers (*Arg1* and *Retnla)* in *LysM*-Cre WT, Drp1$^{fl/fl}$ WT, and MϕKO BMDMs under HSS for 8 h. Data are mean ± SEM. n=3-7. ****p<0.0001, ***p<0.001 (one-way ANOVA followed by Tukey's multiple comparison test). **C.** Cell viability in WT and MϕKO BMDMs exposed to either HSS or normoxia for the indicated time, as measured by a CCK-8-based colorimetric assay**. D.** IF images (left) and quantification (right) of proliferating BrdU+ (green) with nuclei stained by DAPI (blue) after 24 h of HSS exposure in WT and MϕKO BMDMs. Scale bar=20 µm. Data represent n=3-4 independent experiments and are shown as mean ± SEM.

Supplemental Figure S7

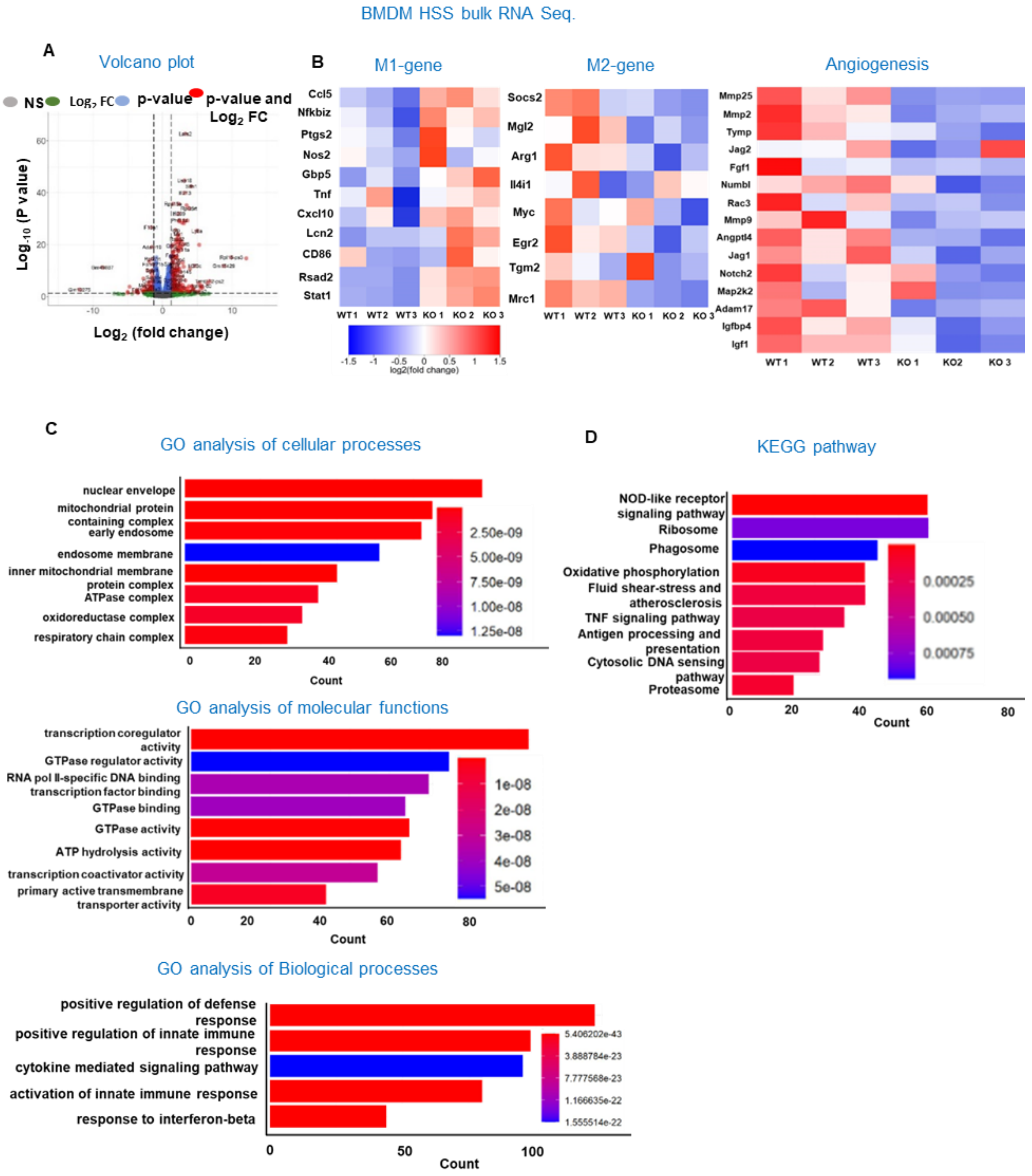

Figure S7: *Drp1*^{KO} BMDM exposed to HSS exhibited increased expression of pro-inflammatory M1 genes and decreased anti-inflammatory M2 genes and pro-angiogenic genes. RNA-Seq analysis was performed on BMDMs from WT and MϕKO mice (n = 3 per group) that were cultured for 7 days and then exposed to HSS for 8h. **A.** A volcano plot showing the differentially expressed genes. **B.** Heat maps depicting the expression patterns of pro-inflammatory M1 genes, anti-inflammatory M2 genes, and pro-angiogenic genes. **C.** Gene Ontology (GO) enrichment analysis of the differentially expressed genes. **D.** KEGG pathway enrichment analysis of the differentially expressed genes.

Supplemental Figure S8

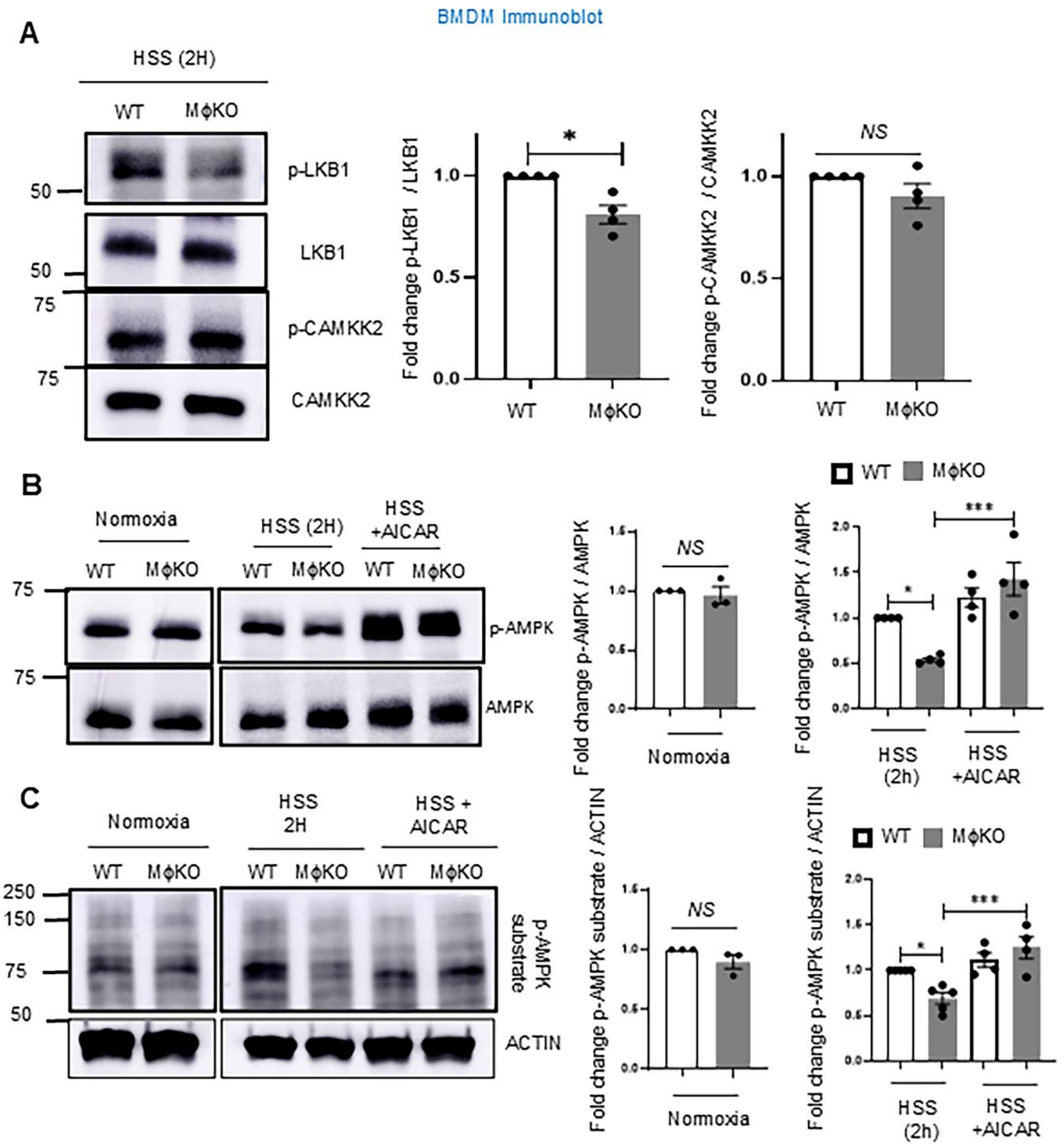

Figure S8: Reduced AMPK activation in *Drp1*^{KO} BMDM under HSS was restored by AMPK activator AICAR. WT and MϕKO BMDMs were subjected to 2 h of HSS stimulation and analyzed by Western blot using anti- p-LKB1, LKB1, p-CAMKK2 and CAMKK2 antibodies. Right panels show quantification. Data are mean ± SEM. (n=4, unpaired t test). **B.** WT and MϕKO BMDMs were exposed to normoxia or HSS for 2 h with or without AICAR pre-treatment (100 μM for 2h) and analyzed by Western blot using anti-p-AMPK, AMPK, total p-AMPK substrate, and ACTIN antibodies. Right panels show quantification. Data are mean ± SEM. n=4-5. (ANOVA followed by Tukey's multiple comparison test).

Supplemental Figure S9

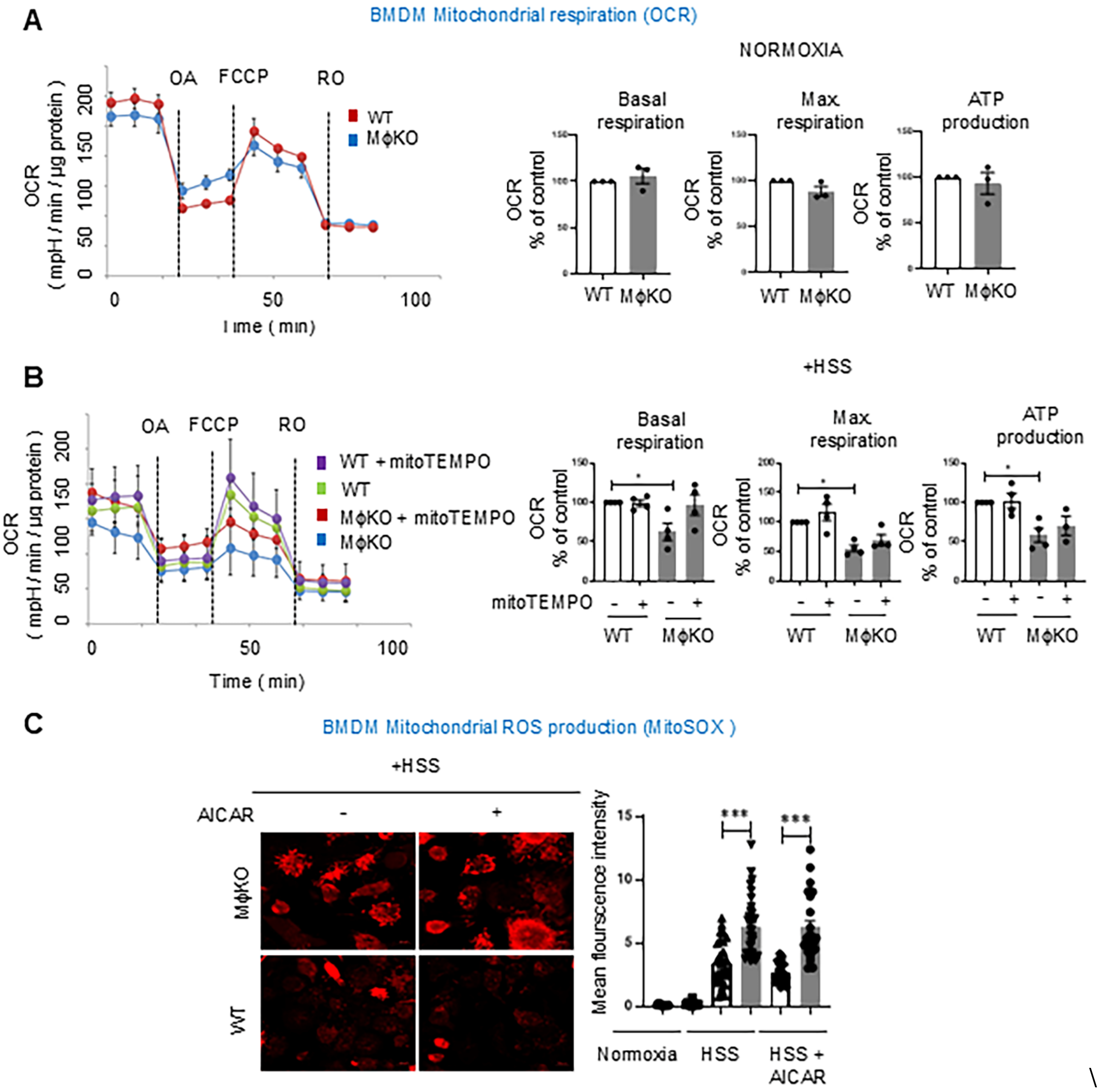

Figure S9: Excess mitoROS production in *Drp1*^{KO} BMDMs under HSS does not precede mitochondrial dysfunction and is not suppressed by the AMPK activator. A. Mitochondrial respiration (OCR) measured via Seahorse XF analyzer in WT and MϕKO BMDMs under normoxia for 2h. **B.** OCR in WT and MϕKO BMDMs after 2h of HSS stimulation, with or without mitoTEMPO pre-treatment (16 h at 20 μM). Right panels show quantification. Data represent n=3 (one-way ANOVA followed by Tukey's multiple comparison test). **C.** Left, Mitochondrial ROS production measured by MitoSOX after 2 h of HSS stimulation in WT and MϕKO BMDMs, with or without AMPK activator AICAR pre-treatment (2 h at 100 μM). Right: Quantification of mean fluorescence intensity using ImageJ. Scale bars=5μm. *p<0.05, ***p<0.001 (one-way ANOVA followed by Tukey's multiple comparison test). Data are mean ± SEM.
