# Supplementary material for: Myeloid DRP1 deficiency limits revascularization in ischemic muscles via inflammatory macrophage polarization and metabolic reprogramming
Source: JCI Insight. 2025 Jan 9;10(1):e177334. doi: 10.1172/jci.insight.177334 (PMC11721294; doi:10.1172/jci.insight.177334)

Fig. 1C Generation of myeloid-specific Drp1^{KO} mice

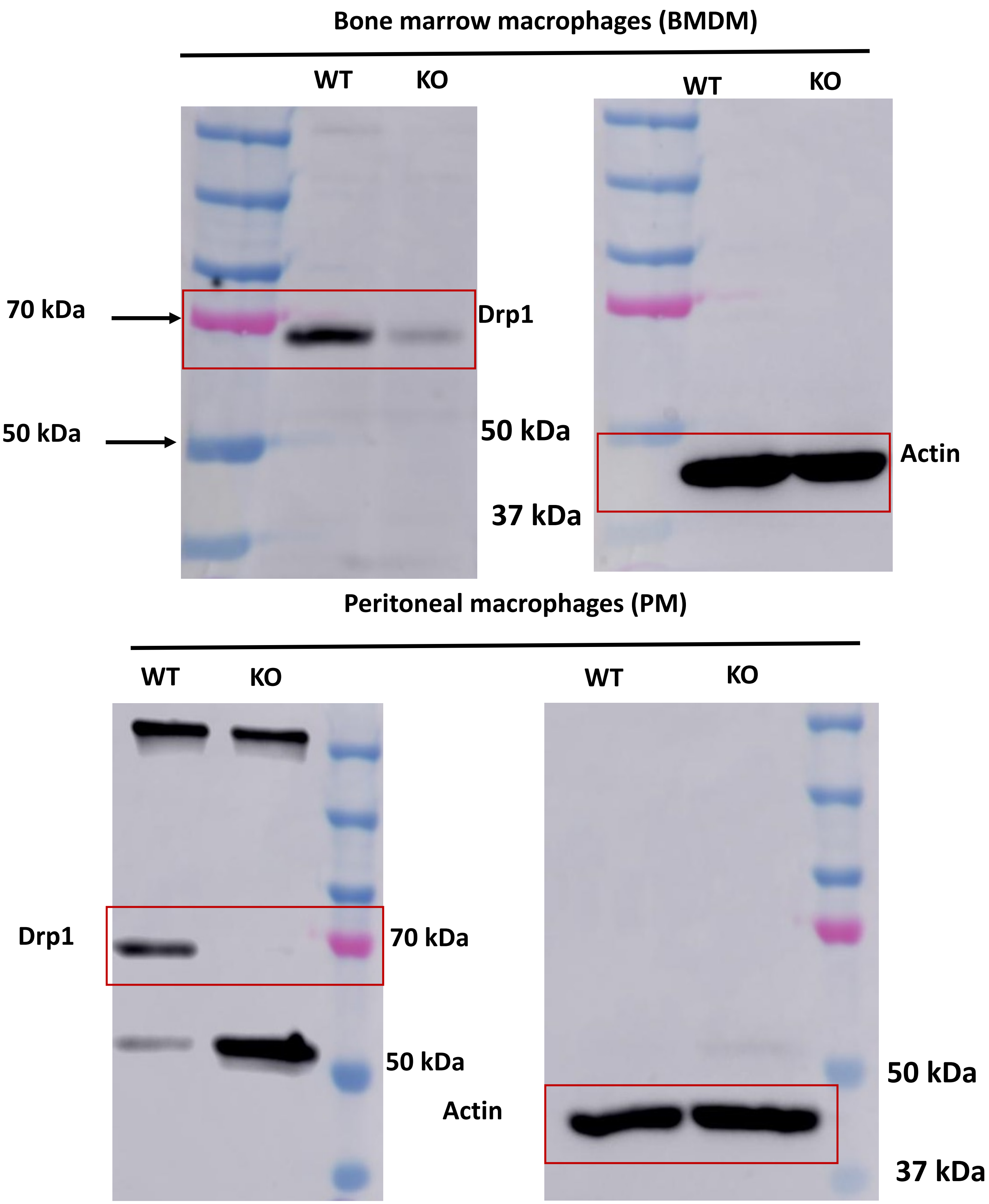

Fig. 1C Generation of myeloid-specific Drp1^{KO} mice

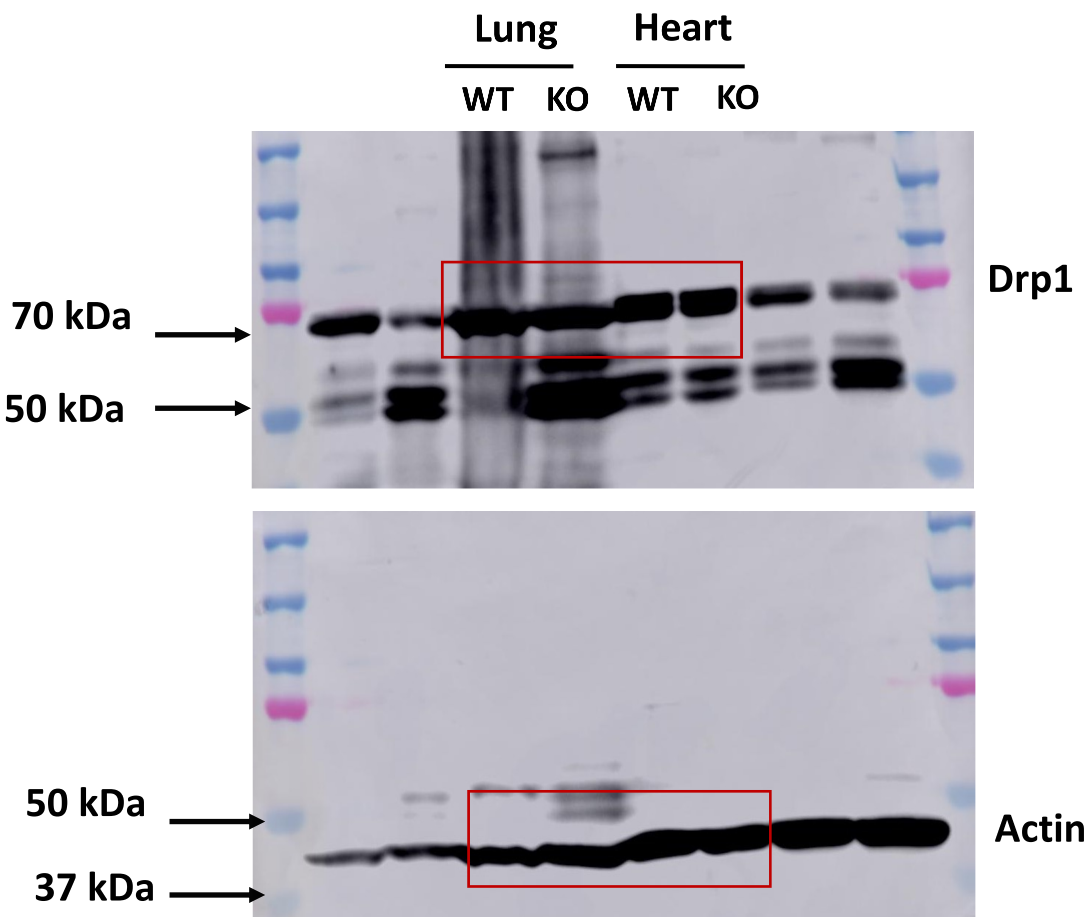

Fig. 3B Ischemic muscle immunoblot

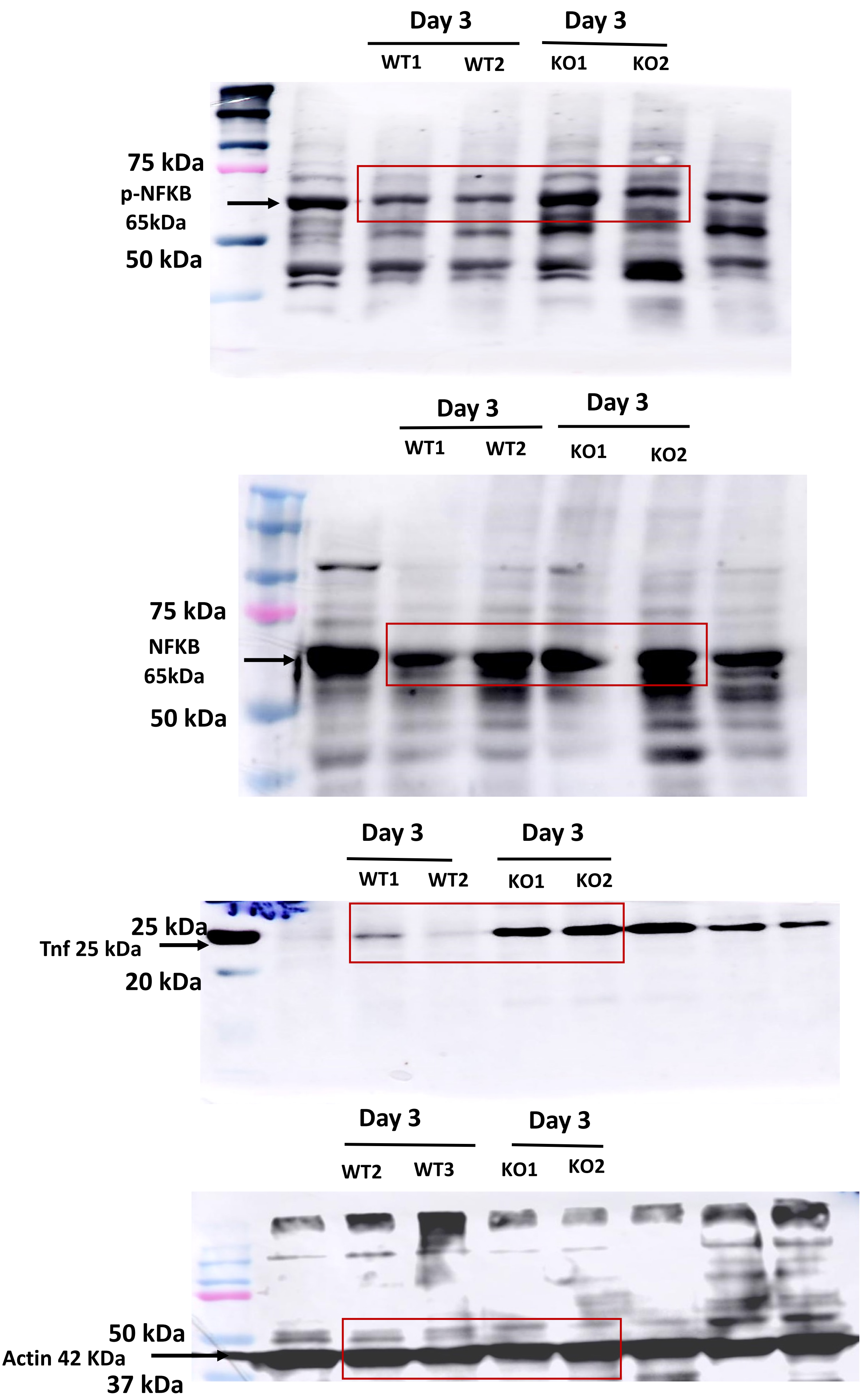

Fig. 3C Ischemic muscle immunoblot

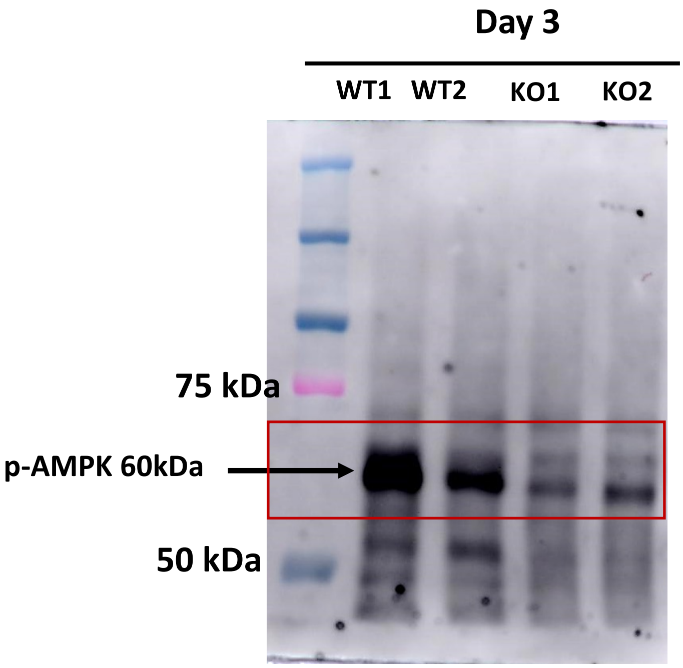

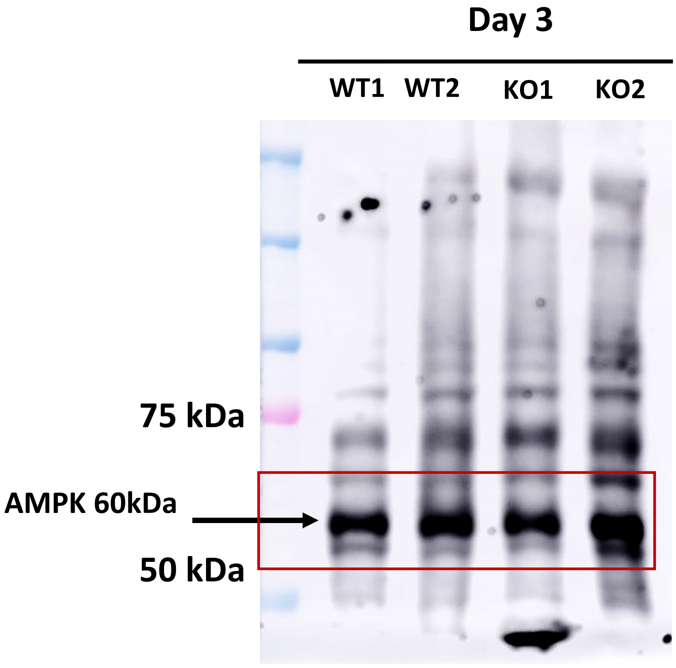

Fig. 4D BMDM Immunoblot

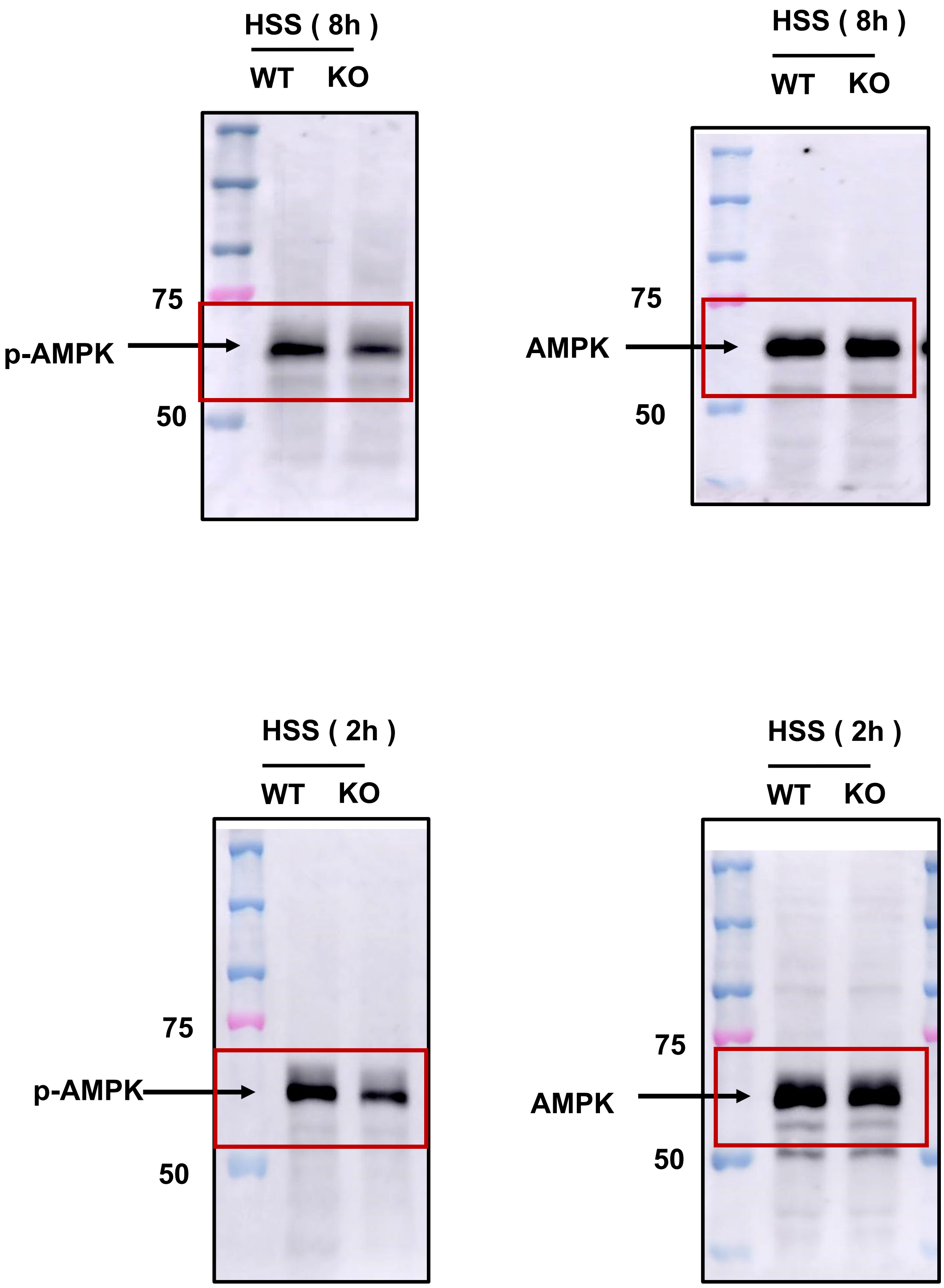

Fig. 6D BMDM HSS CM immunoblot

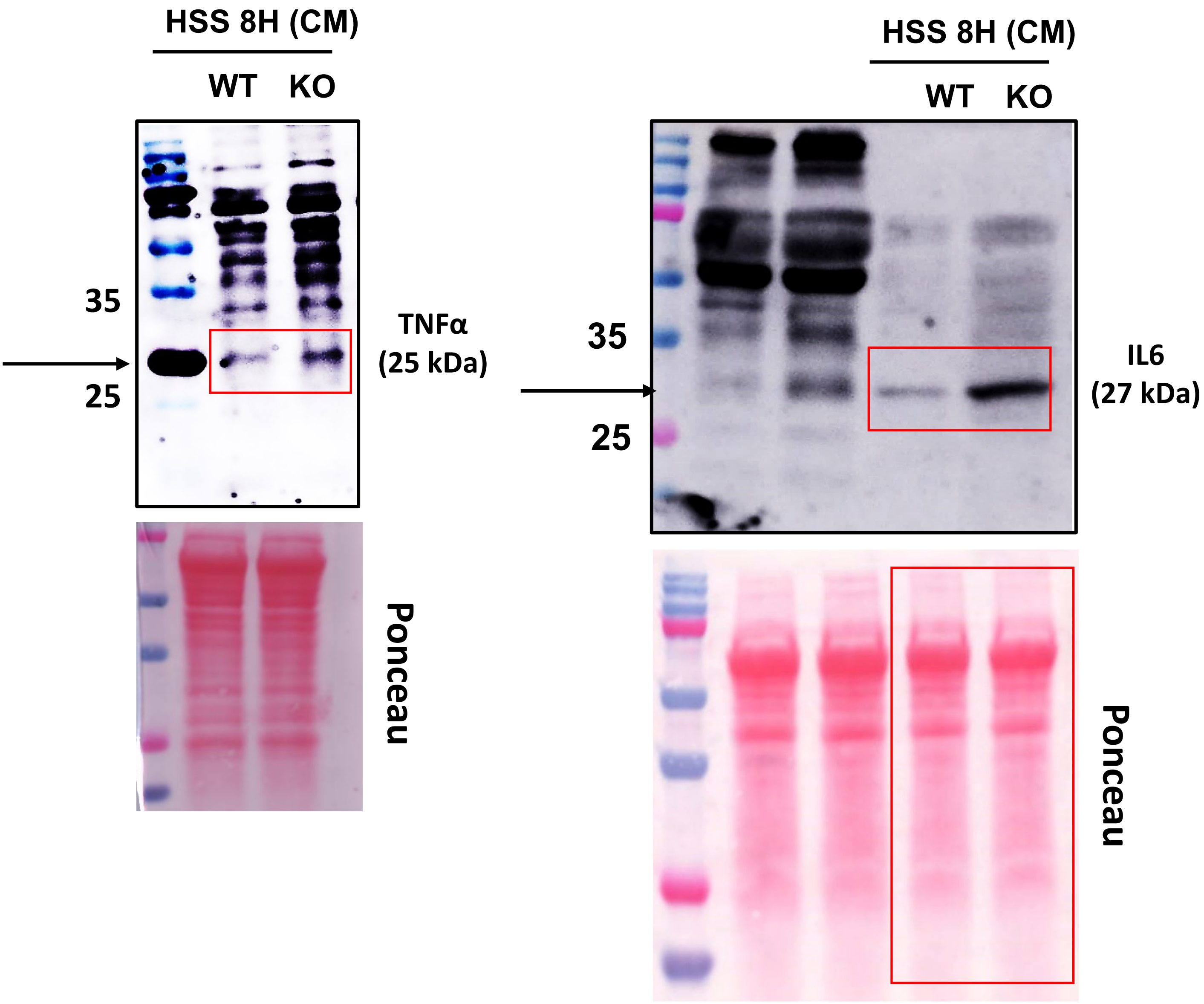

Fig. S5B BMDM Immunoblot

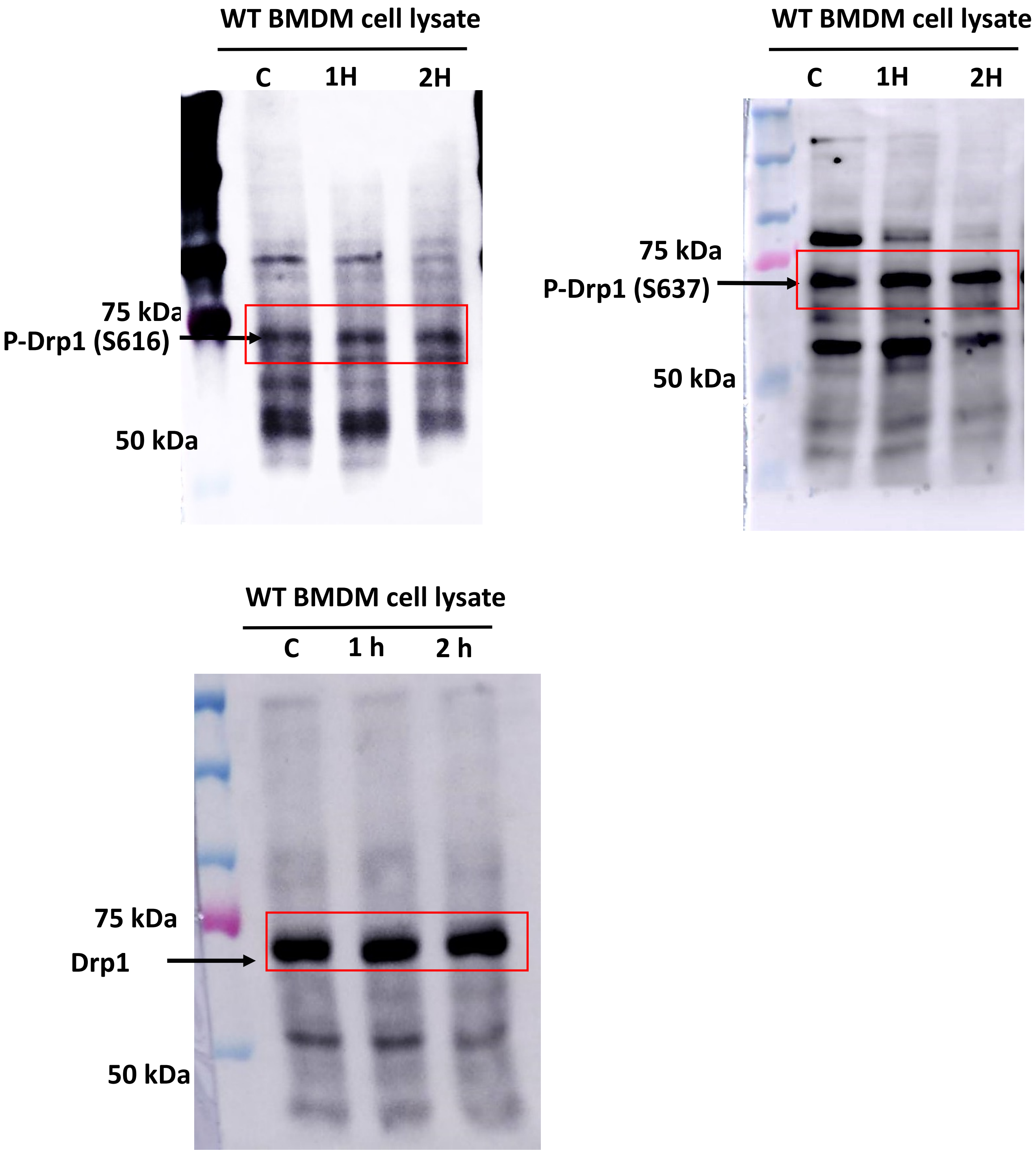

Fig. S5B BMDM Immunoblot

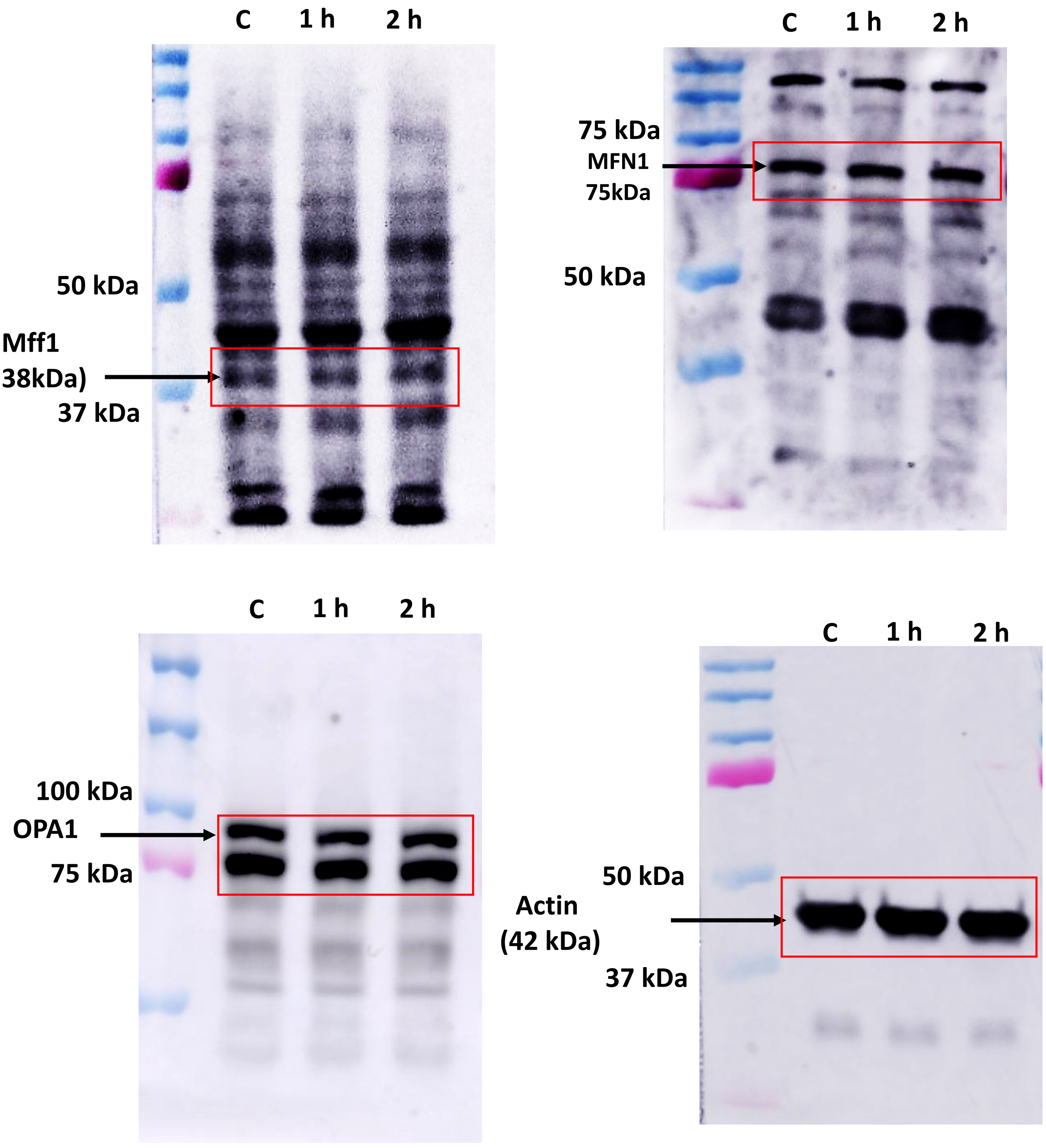

Fig. S8A BMDM Immunoblot

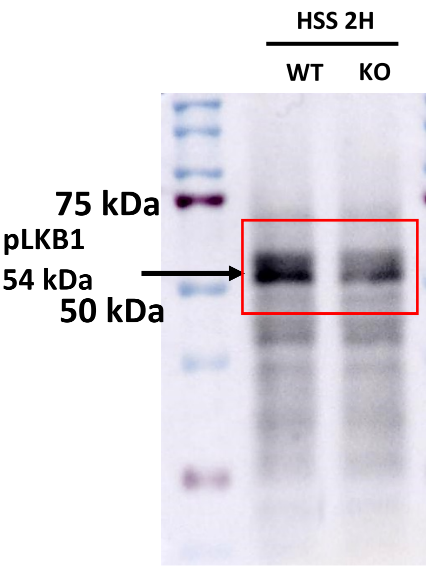

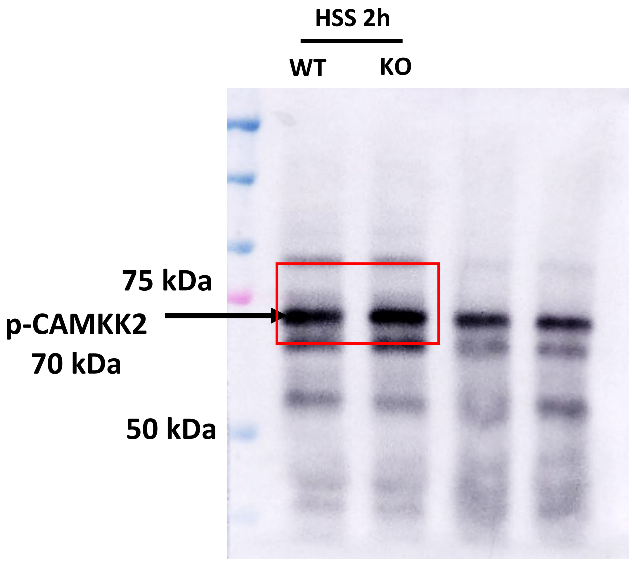

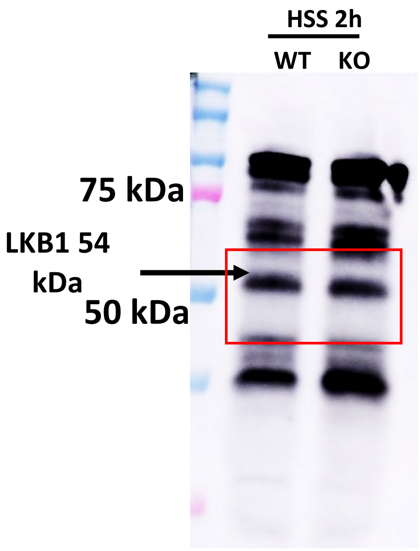

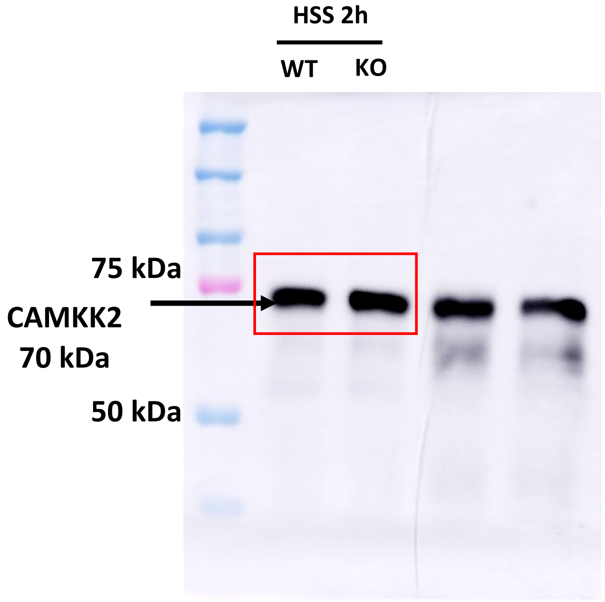

Fig. S8B BMDM Immunoblot

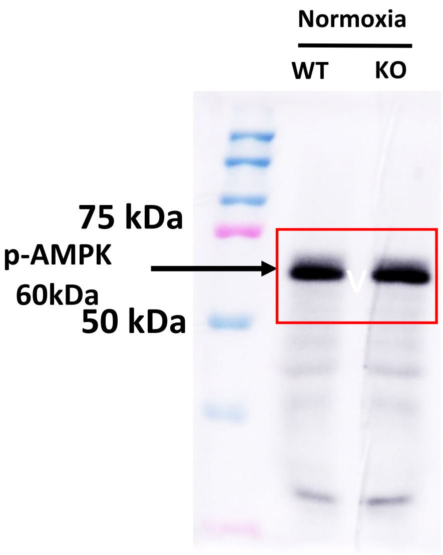

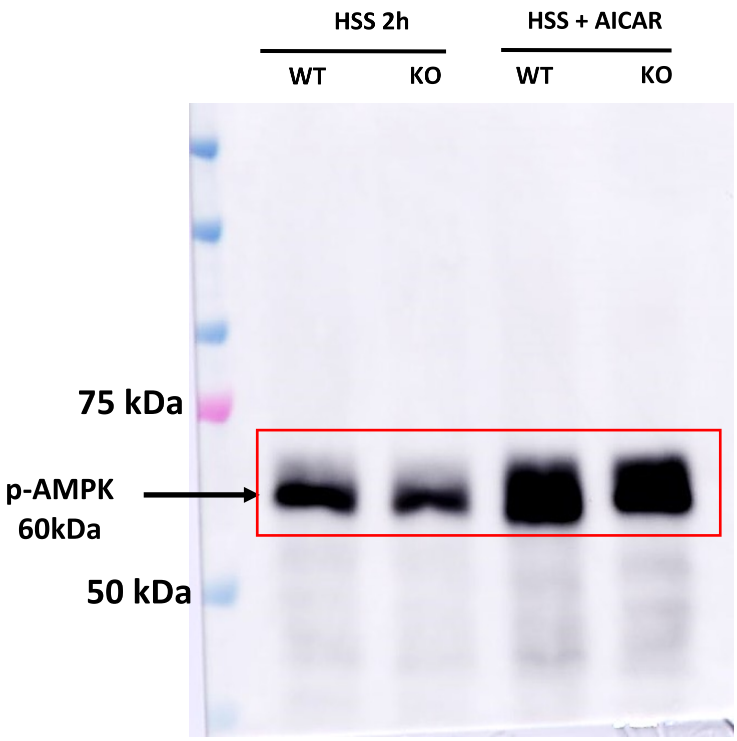

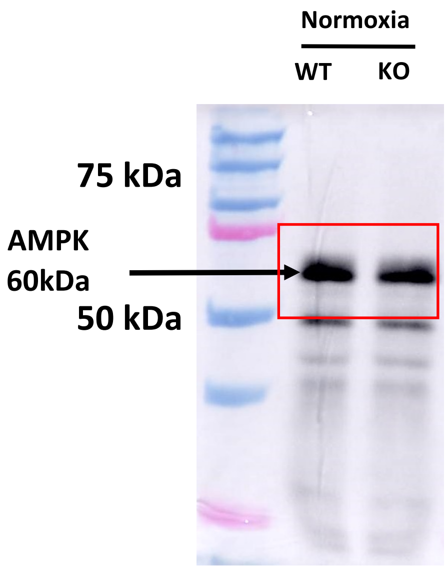

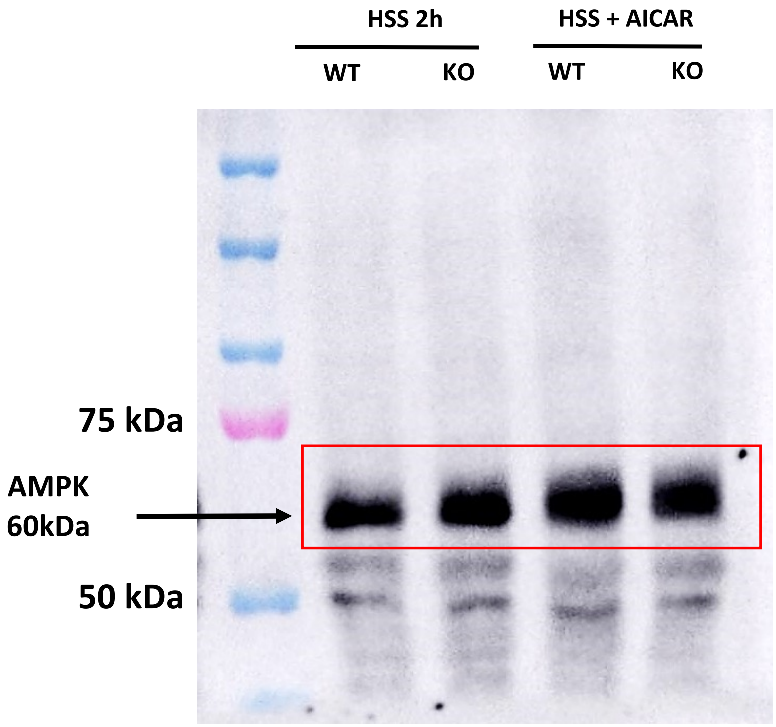

Fig. S8C BMDM Immunoblot

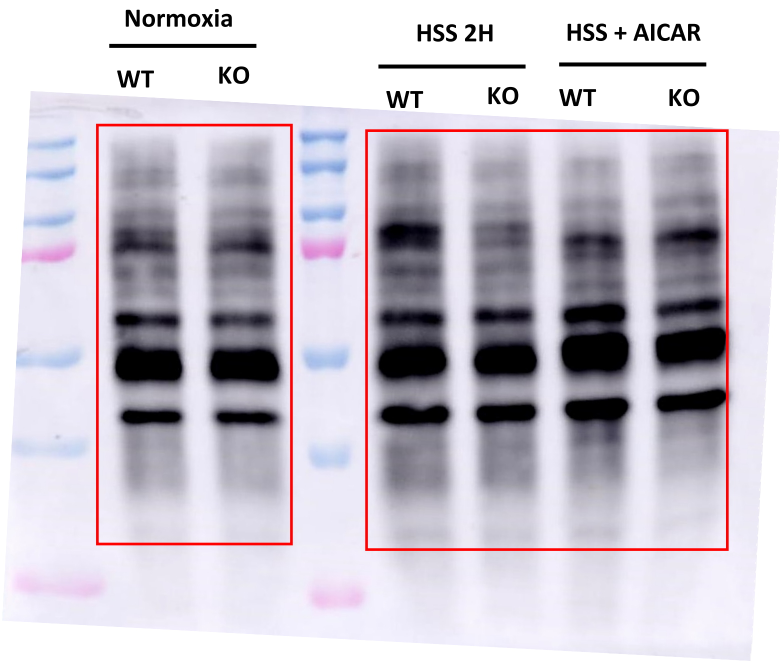

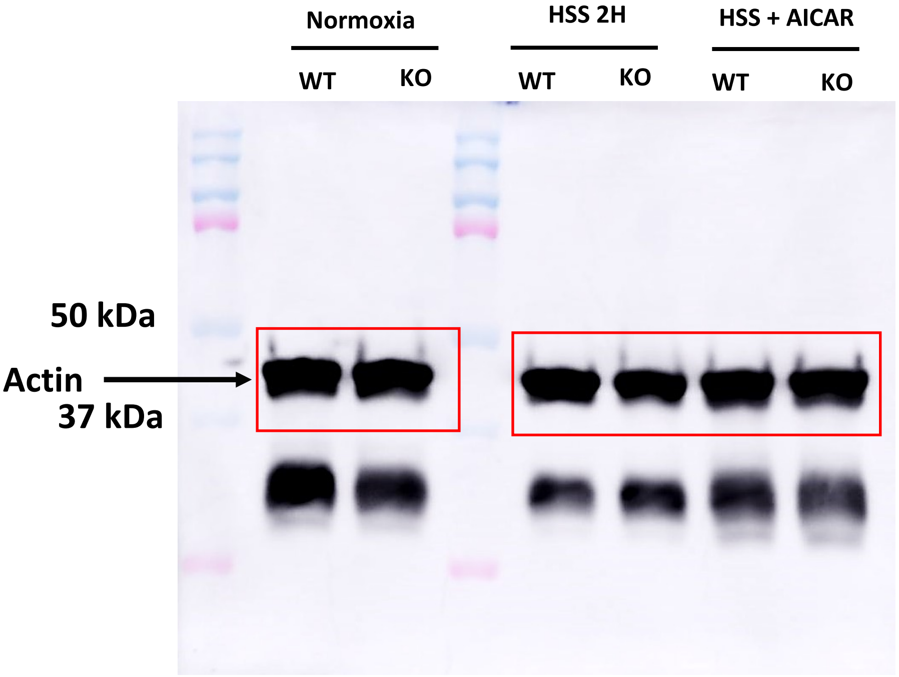

Supplement: Unedited blot and gel images [file jciinsight-10-177334-s093.pdf]
